# The Tryptophan–Kynurenine Pathway as a Key Mediator of the Gut–Brain Axis in Depression and Alzheimer’s Disease

**DOI:** 10.3390/ijms27188122

**Published:** 2026-09-12

**Authors:** Lucia Maria Procopciuc, Adriana Corina Hangan, Sidonia Gog-Bogdan, Roxana Liana Lucaciu

**Affiliations:** 1 Department of Medical Biochemistry, “Iuliu-Hațieganu” University of Medicine and Pharmacy, 400349 Cluj-Napoca, Romania; lprocopciuc@umfcluj.ro; 2 Department of Inorganic Chemistry, Faculty of Pharmacy, “Iuliu-Hațieganu” University of Medicine and Pharmacy, 400012 Cluj-Napoca, Romania; 3 Department of Sugery and ATI, Faculty of Veterinary Medicine, University of Agricultural Sciences and Veterinary Medicine, 400372 Cluj-Napoca, Romania; sidonia.bogdan@usamvcluj.ro; 4 Department of Pharmaceutical Biochemistry and Clinical Laboratory, Faculty of Pharmacy, “Iuliu-Hațieganu” University of Medicine and Pharmacy, 400349 Cluj-Napoca, Romania; liana.lucaciu@umfcluj.ro

**Keywords:** tryptophan metabolism, gut microbiota, kynurenine pathway, neuroinflammation, major depressive disorder, Alzheimer’s disease, intestinal dysbiosis

## Abstract

Depression and Alzheimer’s disease are among the most prevalent and disabling disorders worldwide, imposing a substantial social, economic, and healthcare burden. Increasing evidence suggests that these conditions share several pathophysiological mechanisms, including chronic inflammation, oxidative stress, mitochondrial dysfunction, altered neurotransmission, and gut microbiota dysbiosis. In recent years, the microbiota–gut–brain axis has emerged as a key regulatory system linking gastrointestinal, immune, metabolic, and neural functions. Within this complex network, the tryptophan–kynurenine pathway has gained considerable attention as a critical mediator connecting gut microbial activity, immune responses, and central nervous system function. This review examines the role of the microbiota–gut–brain axis and the tryptophan–kynurenine pathway in the development and progression of depression and Alzheimer’s disease. Particular emphasis is placed on the regulation of tryptophan metabolism by gut microbiota, the inflammatory activation of indoleamine 2,3-dioxygenase, and the generation of neuroactive kynurenine metabolites. Dysregulation of these processes may reduce serotonin synthesis while promoting the accumulation of neurotoxic compounds such as quinolinic acid and 3-hydroxykynurenine, thereby contributing to neuroinflammation, excitotoxicity, cognitive decline, and depressive symptoms. Current evidence also highlights the therapeutic potential of interventions targeting gut microbiota composition and kynurenine pathway activity. A better understanding of these interconnected mechanisms may facilitate the identification of novel biomarkers and the development of personalized strategies for the prevention and treatment of both psychiatric and neurodegenerative disorders.

## 1. Introduction

Depression and Alzheimer’s disease (AD) are among the most prevalent and disabling disorders affecting global health, representing major contributors to disability, morbidity, mortality, and healthcare expenditure worldwide. Their burden is expected to increase substantially in the coming decades as a consequence of population aging, increasing life expectancy, and the growing prevalence of chronic diseases [1,2]. Depression currently affects more than 280 million people globally and remains one of the leading causes of disability across all age groups, contributing significantly to reduced quality of life, impaired social functioning, increased morbidity, and premature mortality [1]. The incidence of depressive disorders has continued to rise during recent decades, driven by demographic, socioeconomic, and environmental factors, as well as improved recognition of mental health conditions [3]. Depression is particularly common among older adults, where it has been associated with accelerated biological aging, frailty, reduced resilience to stress, and an increased risk of cognitive impairment and neurodegenerative disorders [4].

Alzheimer’s disease, the most common cause of dementia, accounts for approximately 60–70% of all dementia cases worldwide and currently affects more than 55 million individuals globally [2]. Characterized by progressive cognitive decline, memory impairment, behavioral disturbances, and loss of functional independence, AD represents one of the leading causes of disability and dependency among older adults. Current projections indicate that the number of people living with dementia may increase to approximately 78 million by 2030 and nearly 139 million by 2050, creating an unprecedented burden on healthcare systems, social services, and caregivers worldwide [5]. In addition to direct medical costs, AD imposes substantial emotional, physical, and economic challenges on families and caregivers [6].

Although depression and AD have traditionally been investigated as distinct clinical entities, growing evidence suggests that they are closely interconnected disorders sharing multiple biological mechanisms. Chronic inflammation, oxidative stress, mitochondrial dysfunction, impaired neuroplasticity, vascular abnormalities, hypothalamic–pituitary–adrenal (HPA) axis dysregulation, and alterations in neurotransmitter metabolism have all been implicated in the pathogenesis of both conditions [7,8]. Furthermore, epidemiological studies consistently demonstrate that individuals with a history of depression exhibit a significantly increased risk of developing dementia later in life. Recent longitudinal analyses suggest that depression may increase dementia risk by more than twofold, supporting the hypothesis that depressive disorders may contribute directly to neurodegenerative processes rather than merely represent an early manifestation of cognitive decline [9]. These findings have stimulated considerable interest in identifying common molecular pathways capable of explaining the biological relationship between depression and AD and providing novel therapeutic targets.

Among the most promising concepts emerging in contemporary neuroscience is the gut–brain axis, a complex bidirectional communication network linking the gastrointestinal tract and the central nervous system through neural, endocrine, metabolic, and immune pathways [10]. This integrated system enables continuous communication between the gut and the brain and plays a fundamental role in maintaining physiological homeostasis. Within this framework, the gut microbiota, consisting of trillions of microorganisms inhabiting the gastrointestinal tract, has emerged as a key regulator of host metabolism, immune function, intestinal barrier integrity, and neurochemical signaling [11]. Increasing evidence indicates that disturbances in microbial composition and function, commonly referred to as dysbiosis, may contribute to the development and progression of several neuropsychiatric and neurodegenerative disorders, including depression and AD [12,13].

One of the major mechanisms through which gut microbiota may influence brain function involves the regulation of tryptophan metabolism. Tryptophan serves as a precursor for serotonin, melatonin, and kynurenine metabolites, with the kynurenine pathway representing its predominant route of degradation [14]. Importantly, this pathway is influenced by inflammatory mediators and microbial metabolites, placing it at the intersection of immune regulation, microbial homeostasis, and central nervous system function [15].

Accumulating evidence suggests that dysbiosis-associated immune activation may alter tryptophan metabolism and promote kynurenine pathway dysregulation, thereby contributing to neuroinflammatory and neurochemical alterations relevant to both depression and AD [16,17,18]. These observations have positioned the kynurenine pathway as a potential molecular link between gut microbiota alterations, systemic inflammation, and central nervous system dysfunction.

Gut microbiota alterations have also been associated with pathological processes relevant to AD and with changes in microbial diversity and metabolite production in major depressive disorder [19,20,21]. These observations support the hypothesis that disturbances of the microbiota gut–brain axis (GBA) may represent a shared biological component of psychiatric and neurodegenerative disorders.

Given the increasing prevalence of depression and AD and the limited effectiveness of current therapeutic strategies, identifying shared biological mechanisms underlying these disorders is of considerable clinical and scientific importance. While gut microbiota alterations, tryptophan metabolism, and kynurenine pathway dysregulation have each been extensively investigated, their integration within a common mechanistic framework encompassing both depression and AD remains less clearly defined.

Therefore, the aim of this review is to examine the tryptophan–kynurenine pathway as a key mediator of microbiota–gut–brain communication in depression and AD. Particular emphasis is placed on the mechanisms through which gut dysbiosis, immune activation, and altered tryptophan availability influence kynurenine metabolism, as well as on the shared and disease-specific consequences of this dysregulation. By integrating biochemical mechanisms with available clinical evidence on kynurenine pathway metabolites, this review also explores the potential implications of these interactions for biomarker development and targeted therapeutic strategies.

The conceptual contribution of this review is the proposal of an integrated microbiota–immune–tryptophan model in which the kynurenine pathway is positioned not merely as one of several metabolic alterations associated with depression and AD, but as a dynamic molecular interface linking intestinal dysbiosis to central nervous system dysfunction. In this model, dysbiosis-associated barrier impairment and immune activation promote indoleamine 2,3-dioxygenase (IDO)-dependent redistribution of tryptophan metabolism, shifting the balance between serotonergic availability and neuroactive kynurenine metabolites. We propose that the biological consequences of this shift depend not simply on overall kynurenine pathway activation, but on the relative balance between neuroprotective and neurotoxic metabolites, together with the inflammatory context and disease stage. This framework may help explain how partially shared upstream disturbances can contribute to predominantly affective manifestations in depression and progressive neurodegenerative changes in AD, while also providing a basis for interpreting kynurenine metabolite ratios as context-dependent biomarkers and potential therapeutic targets.

## 2. Literature Search Strategy

This narrative review was based on a literature search conducted in PubMed/MEDLINE, focusing primarily on studies published between January 2015 and August 2026. Earlier landmark studies were also considered when relevant to fundamental biochemical and physiological mechanisms. Search terms included combinations of “gut microbiota”, “gut-brain axis”, “tryptophan metabolism”, “kynurenine pathway”, “serotonin”, “inflammation”, “depression”, “major depressive disorder”, and “Alzheimer’s disease”. Additional relevant publications were identified from the reference lists of selected articles.

Original clinical and preclinical studies, as well as relevant reviews, were selected according to their relevance to the scope of the article, with particular emphasis on the interactions among gut microbiota, tryptophan metabolism, immune signaling, and kynurenine pathway alterations in depression and AD. Given the narrative nature of this review, no systematic-review protocol, formal risk-of-bias assessment, or meta-analysis was applied.

## 3. The Gut–Brain Axis: Structure and Function

### 3.1. Bidirectional Communication Between the Gut and the Brain

GBA is a highly sophisticated bidirectional communication network that connects the gastrointestinal tract and the central nervous system (CNS), enabling continuous exchange of neural, endocrine, immune, and metabolic signals. This complex system involves multiple interconnected components, including the CNS, enteric nervous system (ENS), autonomic nervous system (ANS), HPA axis, immune system, and the gut microbiota [22,23]. Together, these elements form an integrated communication framework that regulates not only gastrointestinal physiology but also cognition, emotional behavior, stress responsiveness, metabolism, and immune homeostasis.

The concept of gut–brain communication has evolved considerably over recent decades. Historically, the brain was regarded as the primary regulator of gastrointestinal function, exerting control over intestinal motility, secretion, absorption, and blood flow through autonomic and neuroendocrine pathways. While this top-down regulation remains essential for maintaining digestive function, advances in neuroscience, microbiology, and systems biology have revealed that communication within the GBA is fundamentally bidirectional. The gastrointestinal tract is now recognized as an active sensory organ capable of transmitting vast amounts of information to the brain through neural, hormonal, immune, and metabolic mechanisms [24]. This paradigm shift has transformed our understanding of the relationship between the gut and the nervous system, highlighting the intestine as a dynamic contributor to neurological and psychological health.

Communication from the brain to the gut is mediated through autonomic, neuroendocrine, immune, and metabolic pathways, which regulate gastrointestinal motility, secretion, permeability, and immune activity. Conversely, intestinal signals inform the brain about nutrient availability, microbial activity, inflammatory status, and metabolic changes, allowing adaptive physiological and behavioral responses [25,26].

Beyond its established effects on gastrointestinal function, brain-to-gut signaling may also modify the composition and functional activity of the intestinal microbiota. Changes in autonomic activity can influence gastrointestinal motility and transit, epithelial permeability, mucus and fluid secretion, local immune responses, and the physicochemical conditions of the intestinal lumen, thereby modifying the ecological environment in which microbial communities reside. Stress-related activation of the HPA axis may further influence intestinal barrier function and mucosal immunity through glucocorticoid-dependent mechanisms, providing an additional route by which central neuroendocrine states can affect microbial composition and function.

Recent experimental evidence suggests that the brain may exert relatively rapid control over intestinal microbial communities. Toledo et al. demonstrated in mice that modulation of hypothalamic agouti-related peptide (AgRP) and proopiomelanocortin (POMC) neuronal activity, as well as central administration of leptin or ghrelin, induced rapid and anatomically specific changes in gut microbiota composition. These alterations occurred within hours and were associated with changes in duodenal neuronal and synaptic pathways and increased sympathetic tone [27]. These findings support a genuinely bidirectional model in which the brain is not merely a recipient of microbiota-derived signals but may actively shape the intestinal microbial ecosystem. However, because these mechanistic findings were obtained in male mice, the extent to which comparable brain-to-microbiota mechanisms operate in humans remains to be established.

Conversely, communication from the gut to the brain occurs through multiple signaling pathways that continuously inform the CNS about the physiological status of the gastrointestinal environment. Sensory information originating from the intestine includes signals related to nutrient availability, microbial activity, immune responses, mechanical distension, and metabolic processes. These signals are transmitted to the brain through neural pathways, circulating hormones, cytokines, and microbial metabolites, allowing the CNS to adapt behavioral and physiological responses accordingly [28,29].

The gut microbiota represents a central modulator of this communication network, mainly through microbial metabolites, immune regulation, and effects on intestinal barrier integrity [14].

Recent evidence suggests that bidirectional communication within the gut–brain axis plays a crucial role throughout the lifespan, from neurodevelopment to aging. During early life, gut microbiota contributes to the maturation of the immune system, blood–brain barrier, and neural circuits involved in emotional and cognitive processing [12]. In adulthood, this communication network supports metabolic balance, stress adaptation, and cognitive function. In older individuals, however, age-related alterations in microbial diversity, immune regulation, and intestinal barrier integrity may contribute to chronic inflammation and neurodegenerative processes, further emphasizing the importance of maintaining gut–brain homeostasis [30].

Disruption of bidirectional gut–brain communication has been implicated in a growing number of pathological conditions. Dysregulation of neural, endocrine, immune, or microbial signaling pathways may lead to persistent inflammation, altered neurotransmitter metabolism, impaired stress responses, and abnormal neuronal activity. Such alterations have been associated with depression, anxiety disorders, autism spectrum disorders, irritable bowel syndrome, inflammatory bowel diseases, Parkinson’s disease, and AD [13,19]. In particular, increasing evidence indicates that chronic gut dysbiosis may promote neuroinflammation and contribute to the pathogenesis of both psychiatric and neurodegenerative disorders through mechanisms involving immune activation, oxidative stress, and altered tryptophan metabolism [18].

The gut–brain axis represents a dynamic and highly integrated communication system that continuously coordinates gastrointestinal, neurological, endocrine, immune, and metabolic functions. The bidirectional nature of this network allows the organism to respond effectively to internal and external stimuli while maintaining physiological homeostasis. A better understanding of the mechanisms underlying gut–brain communication may provide valuable insights into the pathogenesis of numerous diseases and facilitate the development of innovative therapeutic strategies targeting the microbiota–gut–brain axis.

### 3.2. Neural Pathways: The Vagus Nerve and Enteric Nervous System

Neural communication constitutes one of the fastest and most direct mechanisms within the microbiota GBA, allowing rapid transmission of information between the gastrointestinal tract and the CNS.

The principal neural components involved in this communication network are the ENS and the vagus nerve, which together form a highly integrated signaling pathway capable of regulating gastrointestinal, metabolic, immune, and neurobehavioral functions [23,25].

The ENS is an extensive neuronal network embedded within the wall of the gastrointestinal tract and is often referred to as the “second brain” due to its structural complexity and relative functional autonomy. It contains approximately 400–600 million neurons distributed throughout two major plexuses: the myenteric (Auerbach’s) plexus, located between the longitudinal and circular muscle layers and primarily responsible for regulating intestinal motility, and the submucosal (Meissner’s) plexus, which controls secretion, absorption, local blood flow, and epithelial barrier function [31]. Unlike other peripheral nervous structures, the ENS possesses the ability to independently coordinate digestive functions without direct input from the brain, utilizing local sensory neurons, interneurons, and motor neurons organized into complex reflex circuits [32].

Within the intestinal environment, enteric neurons continuously receive information from mechanical, chemical, and microbial stimuli. Mechanoreceptors detect intestinal distension caused by food intake, whereas chemoreceptors respond to nutrients, hormones, microbial metabolites, and inflammatory mediators present in the intestinal lumen [33]. This sensory information is processed locally within enteric circuits and can also be transmitted to the central nervous system, thereby linking gastrointestinal events with behavioral and physiological responses.

The vagus nerve represents the major anatomical connection between the gastrointestinal tract and the brain and serves as the principal neural pathway mediating gut-to-brain communication. As the tenth cranial nerve and the primary component of the parasympathetic nervous system, the vagus nerve innervates most visceral organs, including large portions of the gastrointestinal tract [34]. Approximately 80% of vagal fibers are afferent, carrying sensory information from the gut toward the brain, whereas only 20% are efferent fibers transmitting signals from the brain to peripheral organs [35]. This anatomical organization highlights the predominant role of the vagus nerve as a sensory conduit through which the brain continuously monitors the physiological state of the gastrointestinal tract.

The mechanism of gut-to-brain signaling begins with the detection of luminal stimuli by intestinal epithelial cells, enteroendocrine cells, immune cells, and enteric neurons. These cells respond to nutrients, microbial metabolites, and inflammatory signals by releasing neurotransmitters, hormones, and signaling molecules capable of activating vagal afferent fibers. Once activated, sensory signals travel through the nodose ganglion to the nucleus tractus solitarius (NTS) in the brainstem, which represents the primary central relay station for visceral information [24,33,34,35]. At the level of the NTS, vagal afferent signaling is predominantly excitatory and glutamatergic, allowing visceral sensory information to be integrated within brainstem circuits before being transmitted to higher-order brain regions [33]. NTS output is further shaped by inhibitory GABAergic and modulatory neurotransmitter systems, providing a mechanism through which visceral information can be dynamically integrated according to the physiological state of the organism [33,34]. From the NTS, integrated visceral information is distributed through ascending brainstem and forebrain networks toward regions, including the hypothalamus, amygdala, hippocampus, insular cortex, and prefrontal cortex, thereby influencing autonomic regulation, emotional processing, cognition, memory, appetite, and stress responses [24,34,35].

The glutamatergic nature of central vagal sensory processing is particularly relevant to the kynurenine pathway. Glutamate-dependent signaling within these circuits provides a potential point of interaction with neuroactive kynurenine metabolites, whose effects on glutamatergic neurotransmission are discussed in Section 4.5. In particular, quinolinic acid (QUIN) enhances N-methyl-D-aspartate (NMDA) receptor-mediated excitatory signaling, whereas kynurenic acid (KYNA) limits NMDA receptor-mediated neurotransmission. Consequently, alterations in the balance between these metabolites may influence glutamatergic signaling within central networks involved in the processing of gut-derived information.

Recent studies have demonstrated that gut microbiota can influence vagal activity through several mechanisms. One important pathway involves microbial production of short-chain fatty acids (SCFAs), particularly acetate, propionate, and butyrate. These metabolites interact with G-protein-coupled receptors expressed on enteroendocrine cells and vagal afferent terminals, stimulating neural signaling toward the brain [36]. In addition, certain bacterial species are capable of producing neurotransmitters or neurotransmitter precursors, including serotonin, gamma-aminobutyric acid (GABA), dopamine, and acetylcholine, which may modulate vagal signaling either directly or indirectly [37].

A particularly important mechanism involves enteroendocrine cells, specialized epithelial cells that function as sensory transducers between the intestinal lumen and the nervous system. These cells express receptors capable of detecting nutrients and microbial metabolites and can rapidly release signaling molecules such as serotonin, glucagon-like peptide-1 (GLP-1), peptide YY (PYY), and cholecystokinin (CCK). Upon activation, enteroendocrine cells establish synapse-like connections with vagal afferent fibers, enabling the rapid transmission of information from the gut to the brain. This process allows microbial and dietary signals to influence appetite regulation, emotional behavior, and cognitive processes within seconds [38].

Experimental evidence supporting the importance of vagal signaling has been obtained through vagotomy studies. Several animal studies have shown that the beneficial behavioral effects induced by specific probiotic strains disappear following vagal nerve transection, indicating that an intact vagal pathway is required for microbiota-mediated modulation of brain function [39]. For example, administration of *Lactobacillus* and *Bifidobacterium* species has been associated with reduced anxiety-like and depressive-like behaviors in rodents; however, these effects are abolished when vagal communication is disrupted [11]. Such findings strongly support the role of the vagus nerve as a critical mediator of microbiota–brain interactions.

Although primarily involved in neural communication, the vagus nerve also interacts with endocrine and immune pathways, contributing to the integration of inflammatory and stress-related signals within the microbiota–gut–brain axis [28,30,40,41].

Collectively, the ENS and vagus nerve constitute the main neural communication pathways of the microbiota–gut–brain axis, enabling bidirectional signaling between the intestinal environment and the CNS. Disruption of these pathways may contribute to altered stress responses, mood disturbances, and cognitive dysfunction.

### 3.3. Endocrine and Immune Signaling Pathways

In addition to neural communication, endocrine and immune pathways constitute major components of the microbiota–gut–brain axis. These signaling systems provide slower but more sustained communication between the gastrointestinal tract and the CNS and contribute to the regulation of metabolic, inflammatory, neuroendocrine, and behavioral responses [22,25].

These signaling systems are closely interconnected and play a central role in maintaining homeostasis under physiological conditions while also contributing to the pathogenesis of numerous neuropsychiatric and neurodegenerative disorders when dysregulated.

The HPA axis represents the principal endocrine pathway involved in gut–brain communication and serves as the body’s primary stress-response system. Activation of the HPA axis occurs in response to both psychological stressors and physiological challenges, including infection, inflammation, and metabolic disturbances [42]. The process begins in the hypothalamus, where stress signals stimulate the secretion of corticotropin-releasing hormone (CRH). CRH subsequently acts on the anterior pituitary gland, inducing the release of adrenocorticotropic hormone (ACTH) into the circulation. ACTH then stimulates the adrenal cortex to synthesize and release glucocorticoids, primarily cortisol in humans [43].

Under physiological conditions, cortisol plays an essential role in maintaining energy homeostasis, regulating immune responses, and facilitating adaptation to environmental stressors. However, chronic activation of the HPA axis may have detrimental consequences for both gastrointestinal and neurological health. Sustained elevations in cortisol levels alter intestinal physiology by reducing mucus production, impairing epithelial tight junction integrity, increasing intestinal permeability, and modifying gastrointestinal motility [26]. These changes facilitate the translocation of bacterial products and inflammatory mediators into systemic circulation, thereby promoting immune activation and systemic inflammation.

One of the most important consequences of prolonged HPA-axis activation is its effect on gut microbial composition. Elevated glucocorticoid levels can alter the abundance and diversity of commensal microorganisms, leading to microbial dysbiosis [29]. Several experimental studies have demonstrated that chronic stress is associated with reductions in beneficial bacterial genera such as *Lactobacillus* and *Bifidobacterium*, accompanied by increased growth of potentially pro-inflammatory microbial species [44]. Such alterations may further compromise intestinal barrier integrity and amplify inflammatory signaling, creating a self-perpetuating cycle of stress, dysbiosis, and immune activation.

Importantly, communication within the endocrine arm of the gut–brain axis is bidirectional. While stress-induced hormonal responses influence gut physiology and microbiota composition, microbial communities themselves are capable of modulating HPA-axis activity. Germ-free animal models have provided compelling evidence for this interaction, demonstrating exaggerated HPA-axis responses to stress compared with conventionally colonized animals [45]. Restoration of microbial colonization during early development partially normalizes neuroendocrine function, indicating that gut microbiota play a critical role in the maturation and regulation of stress-response systems [46]. Furthermore, microbial metabolites, including SCFAs, indole derivatives, and tryptophan metabolites, can influence neuroendocrine signaling pathways and contribute to the regulation of cortisol secretion and stress resilience [14].

In addition to its neural signaling functions, the vagus nerve contributes to immune regulation through the cholinergic anti-inflammatory pathway, a neuroimmune circuit that limits excessive inflammatory responses [40,41]. Activation of vagal efferent signaling suppresses the production of pro-inflammatory cytokines through acetylcholine-dependent immune regulation. A key molecular component of this pathway is the α7 nicotinic acetylcholine receptor (α7nAChR), expressed by macrophages and other cytokine-producing immune cells. Activation of α7nAChRs inhibits intracellular pro-inflammatory signaling, including nuclear factor kappa B (NF-κB) activation, thereby reducing the synthesis and release of cytokines such as tumor necrosis factor α (TNF-α), interleukin-1β (IL-1β), and interleukin-6 (IL-6). In the spleen, vagal anti-inflammatory signaling is functionally linked to splenic sympathetic activity and cholinergic immune mechanisms, providing an additional neuroimmune route through which systemic cytokine production can be restrained [40]. Through these mechanisms, the cholinergic anti-inflammatory pathway contributes to the control of systemic inflammation and may indirectly influence neuroinflammatory processes, including microglial responses within the CNS [47]. It should be noted, however, that much of the detailed mechanistic evidence defining the cholinergic anti-inflammatory pathway, particularly the splenic neuroimmune circuitry and α7nAChR-dependent regulation of cytokine production, has been derived from experimental animal models. Although vagally mediated immunomodulatory mechanisms are also relevant to human physiology, the anatomical organization, relative contribution of individual cellular components, and magnitude of these effects may not fully reproduce those characterized in rodents. This anti-inflammatory mechanism may also be relevant to kynurenine pathway regulation because attenuation of pro-inflammatory cytokine signaling may reduce inflammation-driven IDO activation and consequently limit the diversion of tryptophan metabolism toward the kynurenine pathway.

Beyond endocrine communication, the immune system represents another major pathway linking the gut and the brain. The gastrointestinal tract contains the largest immune compartment in the human body, known as the gut-associated lymphoid tissue (GALT), which comprises approximately 70% of all immune cells [48]. This extensive immune network continuously interacts with dietary antigens, commensal microorganisms, and potential pathogens, maintaining a delicate balance between immune tolerance and protective immune responses.

Under healthy conditions, intestinal epithelial cells, mucus layers, antimicrobial peptides, and immune cells work together to preserve barrier integrity and prevent excessive inflammatory activation. Commensal microorganisms contribute significantly to this process by promoting the differentiation of regulatory T cells (Tregs), stimulating the production of anti-inflammatory cytokines such as interleukin-10 (IL-10), and maintaining epithelial homeostasis [49]. Through these mechanisms, gut microbiota play a fundamental role in preventing chronic inflammation and supporting immune tolerance.

When microbial homeostasis is disrupted, however, intestinal barrier dysfunction may develop. Increased intestinal permeability, often referred to as “leaky gut”, allows bacterial components such as lipopolysaccharides (LPS), peptidoglycans, and other microbial-associated molecular patterns (MAMPs) to enter systemic circulation [50]. These molecules are recognized by pattern-recognition receptors, including Toll-like receptors (TLRs), expressed on immune cells. Activation of these receptors initiates intracellular inflammatory cascades involving NF-κB and other signaling pathways, leading to increased production of pro-inflammatory cytokines such as TNF-α, IL-1β, IL-6, and interferon-gamma (IFN-γ) [12].

These circulating cytokines serve as critical messengers between the peripheral immune system and the brain. Several mechanisms enable inflammatory signals to influence CNS function. Cytokines may access brain regions characterized by increased blood–brain barrier permeability, activate endothelial cells lining cerebral blood vessels, stimulate afferent vagal pathways, or induce the production of secondary inflammatory mediators within the brain. As a result, peripheral immune activation can trigger central neuroinflammatory responses involving astrocytes and microglia, the resident immune cells of the CNS. Beyond their systemic effects, inflammatory mediators can influence blood–brain barrier permeability and microglial activation. Reduced vagal tone has been associated with increased systemic inflammation, impaired stress resilience, and enhanced susceptibility to neurodegenerative processes [51]. Conversely, experimental studies have shown that vagus nerve stimulation exerts neuroprotective effects by attenuating neuroinflammation, reducing oxidative stress, and modulating microglial activity [52]. These observations further support the close integration of neural, endocrine, and immune signaling pathways within the gut–brain axis.

Chronic peripheral inflammation promotes microglial activation, resulting in increased production of cytokines and reactive oxygen species (ROS) that contribute to neuronal dysfunction and neurodegeneration [47].

Particularly relevant to the present review is the interaction between inflammatory cytokines and tryptophan metabolism. Pro-inflammatory cytokines, especially IFN-γ, TNF-α, and IL-6, stimulate the activity of IDO, the rate-limiting enzyme of the kynurenine pathway. Activation of IDO redirects tryptophan metabolism away from serotonin synthesis toward kynurenine production, resulting in decreased serotonin availability and increased generation of neuroactive metabolites. Some of these metabolites, including quinolinic acid and 3-hydroxykynurenine, possess neurotoxic properties and have been implicated in depression, cognitive decline, and AD [51]. Consequently, immune-mediated activation of the kynurenine pathway represents one of the most important molecular mechanisms linking gut dysbiosis, systemic inflammation, and neuropsychiatric disorders.

Collectively, endocrine and immune signaling pathways represent fundamental components of the microbiota–GBA. Through dynamic interactions involving the HPA axis, intestinal immune system, inflammatory mediators, and microbial metabolites, these pathways coordinate physiological responses to internal and external stimuli. Dysregulation of endocrine and immune communication may contribute to chronic inflammation, altered neurotransmitter metabolism, and neurodegeneration, highlighting their critical role in the development of depression and AD.

### 3.4. The Role of Gut Microbiota in Maintaining Homeostasis

The gut microbiota has emerged as one of the most important regulators of human health and a central component of the microbiota–gut–brain axis. The human gastrointestinal tract harbors a highly diverse ecosystem composed of trillions of microorganisms, including bacteria, viruses, fungi, archaea, and protozoa, whose collective genome, known as the microbiome, contains substantially more genes than the human genome itself [53]. Although once considered passive commensals involved primarily in digestion, gut microorganisms are now recognized as active participants in numerous physiological processes, including metabolism, immune regulation, neurodevelopment, endocrine signaling, and maintenance of CNS homeostasis [10].

The composition of the gut microbiota is influenced by multiple factors throughout life, including birth mode, diet, antibiotic exposure, age, genetics, and lifestyle [11]. Under healthy conditions, microbial communities maintain a relatively stable and diverse ecosystem that supports host physiological functions. Disruption of this balance, known as dysbiosis, may contribute to gastrointestinal, metabolic, psychiatric, and neurodegenerative disorders [4,22].

One of the most important functions of the gut microbiota is the maintenance of intestinal barrier integrity. The intestinal barrier consists of several interconnected components, including the mucus layer, epithelial cells, tight junction proteins, antimicrobial peptides, and immune cells. Together, these structures form a highly selective barrier that permits nutrient absorption while preventing the entry of pathogens and potentially harmful substances into systemic circulation [50].

Commensal microorganisms contribute directly to the maintenance of this barrier by stimulating mucus production, promoting epithelial cell renewal, and enhancing the expression of tight junction proteins such as occludin, claudins, and zonula occludens-1 (ZO-1) [54]. Several commensal bacterial genera, including *Lactobacillus* and *Bifidobacterium*, have been associated with mechanisms that support epithelial barrier integrity, including metabolite production, competitive exclusion of pathogens, and antimicrobial activity [48]. However, many of the mechanistic effects attributed to individual bacterial taxa have been characterized predominantly in experimental models, and their magnitude and functional consequences in the human gut may depend on strain specificity, microbial community context, diet, and host factors. Therefore, the designation of individual taxa as uniformly “beneficial” should be interpreted cautiously, particularly when extrapolating findings from animal models to humans.

When dysbiosis develops, disruption of epithelial barrier integrity may occur, resulting in increased intestinal permeability and translocation of microbial products into the circulation. This phenomenon may compromise physiological homeostasis and contribute to systemic alterations associated with disease states [12].

Beyond barrier maintenance, the gut microbiota contributes substantially to metabolic homeostasis through the fermentation of dietary fibers into SCFAs, primarily acetate, propionate, and butyrate [36]. These metabolites provide energy for colonocytes, reinforce epithelial integrity, regulate immune responses, and influence glucose and lipid metabolism through G-protein-coupled receptors such as GPR41 and GPR43 [55,56]. SCFAs also participate in gut–brain communication by modulating neurotransmitter synthesis, neuronal plasticity, and metabolic signaling pathways [30].

Among SCFAs, butyrate has attracted particular interest because of its neuroprotective properties. As a histone deacetylase (HDAC) inhibitor, it modulates gene expression and promotes neurotrophic factor production, neuronal plasticity, and cognitive function [28,57].

Another critical role of the gut microbiota involves the regulation of neurotransmitter synthesis and neurochemical homeostasis. Another critical role of the gut microbiota involves the modulation of host neurochemical signaling. In this context, “modulation” encompasses several distinct mechanisms, including direct microbial production of certain neuroactive compounds or their precursors, microbial transformation of substrates and metabolites that influence their local availability, and indirect regulation of host neurotransmitter synthesis, release, or signaling through microbial metabolites and interactions with enteroendocrine, immune, and neural pathways [37]. Thus, microbiota-associated changes in neuroactive systems, including serotonergic, dopaminergic, GABAergic, cholinergic, histaminergic, and noradrenergic signaling, should not necessarily be interpreted as direct microbial synthesis of the corresponding neurotransmitters. Rather, these effects may reflect a combination of direct microbial metabolic activity and indirect modulation of host neurochemical pathways. Because most peripheral neurotransmitters do not readily cross the blood–brain barrier, microbiota-related effects on central neurotransmission may also be mediated through vagal, immune, endocrine, and metabolic signaling mechanisms.

Particularly important in the context of depression and AD is the microbial regulation of tryptophan metabolism. Tryptophan is an essential amino acid that serves as a precursor for serotonin, melatonin, and kynurenine pathway metabolites. Gut microorganisms influence both the availability of dietary tryptophan and the metabolic pathways through which it is processed [14]. These interactions are discussed in detail in Section 5. Under physiological conditions, a balanced microbiota helps maintain appropriate levels of serotonin synthesis and kynurenine metabolism.

The gut microbiota also plays a fundamental role in immune homeostasis. Continuous interactions between commensal microorganisms and intestinal immune cells contribute to the development and maintenance of immune tolerance. Beneficial microbial species stimulate Treg cells, enhance production of anti-inflammatory cytokines such as IL-10, and suppress excessive inflammatory responses [49]. Through these mechanisms, the microbiota prevents chronic immune activation and maintains a balanced inflammatory environment. Dysbiosis may disrupt immune homeostasis and alter host–microbe interactions, contributing to an imbalance between pro-inflammatory and anti-inflammatory signaling pathways [52].

Collectively, the gut microbiota contributes to intestinal, metabolic, immune, and neurological homeostasis through its effects on barrier integrity, metabolite production, neurotransmitter regulation, and host–microbe interactions. Disturbances in microbial composition may therefore influence multiple physiological systems involved in the maintenance of brain health.

## 4. Tryptophan Metabolism: Biochemical Pathways

### 4.1. Sources and Physiological Functions of Tryptophan

Tryptophan is an essential amino acid that plays a fundamental role in human physiology. Because humans lack the enzymatic machinery required for its de novo synthesis, tryptophan must be obtained exclusively through dietary intake [58]. It serves not only as a building block for protein synthesis but also as a precursor for several biologically active molecules involved in neurotransmission, immune regulation, endocrine signaling, and cellular energy metabolism. Consequently, alterations in tryptophan availability or metabolism may have profound effects on both peripheral and central physiological processes [14].

Dietary tryptophan is present in a wide variety of protein-rich foods, including meat, poultry, fish, eggs, milk and dairy products, legumes, soy products, seeds, and nuts. Following ingestion, dietary proteins are digested in the gastrointestinal tract, releasing free amino acids that are absorbed primarily in the small intestine through active transport mechanisms [59]. Once absorbed, tryptophan enters the circulation, where approximately 80–90% is bound to albumin, while the remaining fraction circulates as free tryptophan, which represents the biologically active form available for cellular uptake and metabolic conversion [60].

The physiological availability of tryptophan is influenced not only by dietary intake but also by competition with other large neutral amino acids (LNAAs), including leucine, isoleucine, valine, phenylalanine, and tyrosine. These amino acids share a common transporter system, known as the large amino acid transporter 1 (LAT1), for passage across the blood–brain barrier [61]. Consequently, the ratio of circulating tryptophan to competing LNAAs is considered a major determinant of brain tryptophan availability and subsequent neurotransmitter synthesis. Changes in dietary composition, metabolic status, or inflammatory conditions may therefore influence CNS tryptophan concentrations and alter downstream metabolic pathways [62].

Beyond its role in protein synthesis, tryptophan serves as the precursor for three major metabolic pathways: the serotonin pathway, the kynurenine pathway, and the microbial indole pathway. Under physiological conditions, approximately 90–95% of dietary tryptophan is metabolized through the kynurenine pathway, while only a small proportion is converted into serotonin and melatonin [15]. Despite accounting for a relatively minor fraction of total tryptophan metabolism, serotonin synthesis has profound implications for mood regulation, cognition, sleep, appetite, and gastrointestinal function. In contrast, the kynurenine pathway plays a central role in immune regulation, neuroinflammation, and the synthesis of nicotinamide adenine dinucleotide (NAD^+^), an essential cofactor involved in cellular energy metabolism [17]. Tryptophan is the precursor of serotonin and melatonin, molecules involved in mood regulation, cognition, and circadian rhythm control [63,64,65].

In addition to its neurological functions, tryptophan plays an important role in immune regulation. Immune cells actively metabolize tryptophan through the kynurenine pathway as part of physiological mechanisms that control inflammation and immune tolerance. Depletion of local tryptophan concentrations and generation of kynurenine metabolites contribute to the modulation of T-cell proliferation, macrophage activity, and cytokine production [66]. This relationship highlights the intimate connection between amino acid metabolism and immune homeostasis.

Gut microbiota influences tryptophan availability and metabolizes tryptophan into bioactive indole derivatives that regulate intestinal barrier function and immune signaling through receptors such as AhR [67,68].

Another important physiological function of tryptophan involves cellular energy production through the kynurenine pathway. The terminal steps of this pathway generate NAD^+^, an essential coenzyme required for mitochondrial respiration, ATP synthesis, DNA repair, and regulation of cellular stress responses [60]. Given the central role of mitochondrial dysfunction in aging and neurodegenerative diseases, disturbances in tryptophan metabolism may have significant implications for neuronal survival and cognitive function.

Altogether, tryptophan represents far more than a simple dietary amino acid. As a precursor of serotonin, melatonin, kynurenine metabolites, microbial indoles, and NAD+, it occupies a central position at the intersection of neurobiology, immunology, metabolism, and microbiome research. Understanding the physiological functions and metabolic fate of tryptophan is therefore essential for elucidating the molecular mechanisms linking gut microbiota, depression, aging, and AD.

### 4.2. The Serotonin Pathway

The serotonin pathway represents one of the principal metabolic fates of tryptophan and plays a critical role in the regulation of mood, cognition, emotional behavior, sleep, appetite, gastrointestinal function, and neuroendocrine activity. Although only approximately 1–5% of dietary tryptophan is converted into serotonin, this pathway exerts profound physiological effects due to the diverse functions of serotonin as both a neurotransmitter and a peripheral signaling molecule [14,63].

Serotonin, also known as 5-hydroxytryptamine (5-HT), is synthesized through a two-step enzymatic process. The biosynthesis of serotonin begins with the hydroxylation of tryptophan to 5-hydroxy-L-tryptophan (5-HTP), a reaction catalyzed by tryptophan hydroxylase (TPH), which represents the rate-limiting step of the pathway. Subsequently, 5-HTP is decarboxylated by aromatic L-amino acid decarboxylase (AADC), resulting in the formation of serotonin [69].

Two isoforms of tryptophan hydroxylase have been identified. Tryptophan hydroxylase 1 (TPH1) is predominantly expressed in enterochromaffin cells of the gastrointestinal tract and is responsible for peripheral serotonin synthesis, whereas tryptophan hydroxylase 2 (TPH2) is mainly expressed in serotonergic neurons within the brainstem and mediates central serotonin production [70]. This distinction is physiologically important because serotonin itself cannot readily cross the blood–brain barrier. Consequently, central and peripheral serotonin pools are largely independent and are regulated through distinct mechanisms [60].

Approximately 90–95% of total serotonin in the human body is synthesized by enterochromaffin cells located within the intestinal mucosa [71]. In the gastrointestinal tract, serotonin serves as a key regulator of intestinal motility, secretion, visceral sensitivity, and communication with the ENS. Mechanical stimulation of the intestinal wall or exposure to luminal nutrients induces serotonin release, which subsequently activates intrinsic enteric neurons and vagal afferent pathways [72]. Through these mechanisms, intestinal serotonin contributes to digestive regulation and indirectly participates in gut–brain communication through enteric and vagal signaling rather than by directly entering the CNS.

Within the CNS, serotonergic neurons are primarily localized in the raphe nuclei of the brainstem. These neurons project extensively throughout the brain, including the hippocampus, amygdala, hypothalamus, and prefrontal cortex, regions critically involved in emotional regulation, cognition, memory formation, reward processing, and stress adaptation [73]. Through interactions with multiple serotonin receptor subtypes, serotonin modulates neuronal excitability, synaptic plasticity, and neurotransmitter release, thereby influencing a broad spectrum of behavioral and cognitive functions.

Serotonergic dysfunction has long been implicated in depression and remains an important therapeutic target despite the multifactorial nature of the disorder [64,74].

Serotonin plays an important role in neurogenesis, synaptic plasticity, and adaptive neuronal responses, while altered serotonergic signaling may contribute to impaired cognitive and emotional function [75,76,77].

Serotonin also participates in the regulation of appetite and energy homeostasis. Within hypothalamic circuits, serotonergic signaling influences satiety mechanisms, food intake, and energy expenditure. Dysregulation of these pathways has been associated with obesity, metabolic syndrome, and eating disorders, highlighting the broader physiological significance of serotonin beyond its neurological functions [78].

An additional aspect of serotonin metabolism involves its conversion into melatonin. Within the pineal gland, serotonin is first converted into N-acetylserotonin by the enzyme arylalkylamine N-acetyltransferase (AANAT). N-acetylserotonin is subsequently methylated by acetylserotonin O-methyltransferase (ASMT), resulting in the formation of melatonin [79]. Through this metabolic pathway, tryptophan indirectly contributes to the regulation of circadian rhythms, sleep–wake cycles, antioxidant defense mechanisms, and neuroprotection.

Growing evidence indicates that the gut microbiota can influence peripheral serotonin biosynthesis, although the underlying mechanisms and the strength of the evidence differ between experimental and human studies. Mechanistic studies, particularly in animal and experimental models, have shown that microbial metabolites, including SCFAs, can stimulate tryptophan hydroxylase 1 (TPH1) expression in enterochromaffin cells and thereby enhance intestinal serotonin production [80]. Several bacterial taxa, including members of the genera *Bifidobacterium*, *Lactobacillus*, *Clostridium*, and *Enterococcus*, have also been associated with modulation of serotonergic signaling and peripheral serotonin concentrations [71]. However, direct extrapolation of these findings to humans requires caution because microbial effects may be species-, strain-, and host-dependent. Moreover, microbiota-induced changes in intestinal serotonin should not be interpreted as direct changes in central serotonin concentrations, since peripheral and central serotonin pools are largely separated by the blood–brain barrier and are regulated through distinct mechanisms.

The gut microbiota may also affect serotonin metabolism by regulating tryptophan availability. Through competition for dietary tryptophan, modulation of intestinal absorption, and interactions with host metabolic pathways, microbial communities can influence the amount of substrate available for serotonin synthesis [67]. Consequently, alterations in microbial composition may contribute to disturbances in serotonergic neurotransmission through both direct and indirect mechanisms.

Overall, serotonin represents an important link between tryptophan metabolism and gastrointestinal, neuroendocrine, and CNS function. Its synthesis is influenced by tryptophan availability, gut microbiota, inflammatory processes, and neuroendocrine signaling, supporting its relevance within the microbiota–gut–brain axis.

**Figure 1:**
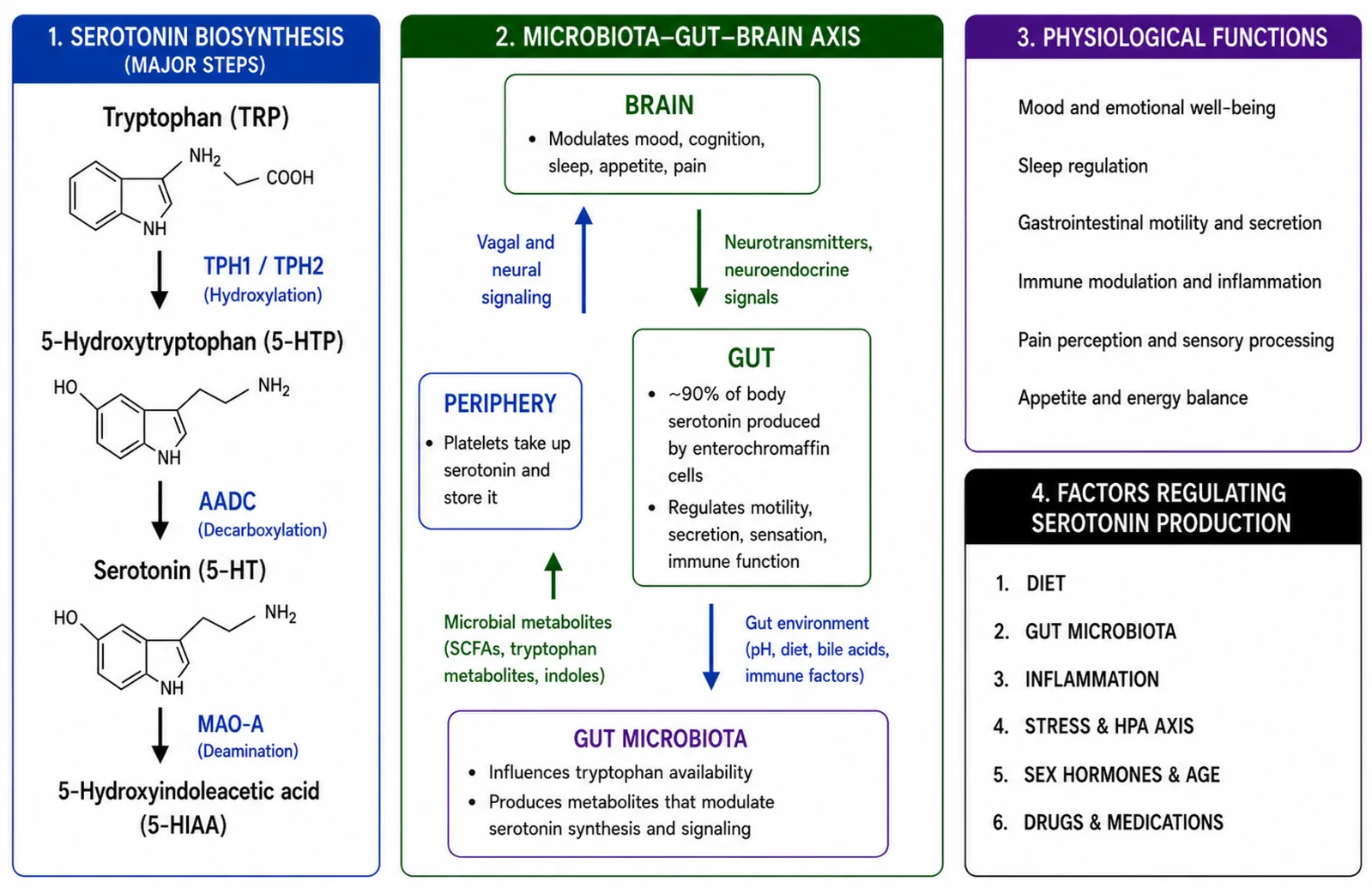
**Serotonin biosynthesis and its regulation within the microbiota–gut–brain axis.** Tryptophan is converted to 5-HT through the sequential formation of 5-HTP, catalyzed by TPH1/TPH2, followed by decarboxylation mediated by AADC. Serotonin is subsequently metabolized by MAO-A to 5-HIAA. Within the microbiota–gut–brain axis, bidirectional communication between the gut and brain involves neural, neuroendocrine, and microbial signals, while gut microbiota-derived metabolites modulate tryptophan availability and serotonergic signaling. Serotonin contributes to multiple physiological functions, including mood, sleep, gastrointestinal function, immune regulation, pain perception, and appetite. Its production and signaling are influenced by diet, gut microbiota, inflammation, the stress/HPA axis, sex hormones, age, and pharmacological agents. **Abbreviations:** 5-HIAA, 5-hydroxyindoleacetic acid; 5-HT, serotonin (5-hydroxytryptamine); 5-HTP, 5-hydroxytryptophan; AADC, aromatic L-amino acid decarboxylase; HPA, hypothalamic–pituitary–adrenal; MAO-A, monoamine oxidase A; SCFAs, short-chain fatty acids; TPH, tryptophan hydroxylase.

The major steps of serotonin biosynthesis, its physiological functions, and the factors regulating its production within the microbiota–gut–brain axis are summarized in Figure 1.

### 4.3. The Kynurenine Pathway: Overview and Regulation

The kynurenine pathway constitutes the principal route of tryptophan degradation in mammals and represents one of the most important metabolic interfaces between the immune system, the CNS, and the gut microbiota. Under physiological conditions, approximately 90–95% of dietary tryptophan is metabolized through this pathway, whereas only a small proportion is utilized for serotonin and melatonin synthesis [14,15]. Although initially considered a pathway primarily involved in amino acid catabolism, extensive research conducted over the past two decades has demonstrated that kynurenine metabolism plays a crucial role in regulating immune responses, redox balance, neurotransmission, neuroplasticity, and cellular energy homeostasis [17].

The kynurenine pathway begins with the oxidative cleavage of the indole ring of tryptophan, generating N-formylkynurenine, an unstable intermediate that is rapidly converted into kynurenine. This initial step represents the rate-limiting phase of the entire pathway and serves as a major regulatory checkpoint controlling the distribution of tryptophan between serotonin synthesis and kynurenine metabolism [81]. Consequently, increased kynurenine pathway activity may reduce tryptophan availability for serotonin synthesis, thereby influencing the metabolic balance between these two major pathways.

Kynurenine occupies a central position within the pathway because it functions as a branching point from which several biologically active metabolites can be generated. Once formed, kynurenine is distributed throughout the body and can readily cross the blood–brain barrier via large neutral amino acid transporters. This property is particularly important because peripheral alterations in tryptophan metabolism may directly influence brain biochemistry and neuronal function [16]. As a result, activation of the kynurenine pathway in peripheral tissues can exert significant effects on the CNS even in the absence of local brain inflammation.

Following its formation, kynurenine may enter two major metabolic branches that differ substantially in their biological consequences. One branch leads predominantly to the production of neuroprotective metabolites, whereas the other generates compounds that may exert neurotoxic and pro-inflammatory effects under pathological conditions [17].

These branches generate neuroactive metabolites, including KYNA, 3-hydroxykynurenine (3-HK), and QUIN, whose biological effects are discussed in detail in Section 4.5 [82,83,84].

The biological consequences of kynurenine pathway activation depend on the balance among its downstream metabolites. This balance is dynamically regulated and may be altered by inflammatory, metabolic, and environmental factors, with potential consequences for neuronal homeostasis [64].

Inflammation represents an important regulator of kynurenine pathway activity. Pro-inflammatory cytokines can enhance tryptophan degradation and kynurenine formation, and inflammatory conditions are therefore frequently associated with an increased kynurenine-to-tryptophan (KYN/TRP) ratio, commonly used as an indirect marker of pathway activation [66,85].

This inflammatory regulation has important implications for neuropsychiatric disorders. Increased kynurenine pathway activity reduces serotonin availability while simultaneously increasing the generation of potentially neurotoxic metabolites. Thus, chronic inflammation may contribute to depressive symptoms through both serotonergic deficiency and accumulation of neuroactive kynurenine derivatives [86].

Within the CNS, kynurenine metabolism exhibits marked cellular specialization. Astrocytes preferentially produce kynurenic acid because they possess limited capacity to generate downstream neurotoxic metabolites. Conversely, activated microglia are the primary source of QUIN and other neurotoxic kynurenine derivatives [87]. Under conditions of chronic neuroinflammation, microglial activation may therefore shift the pathway toward excessive QUIN production, amplifying oxidative stress and excitotoxic injury.

In addition to inflammation, stress-related neuroendocrine mechanisms also regulate kynurenine metabolism. Activation of the HPA axis increases glucocorticoid secretion, which promotes tryptophan degradation through stress-responsive metabolic pathways [44]. Chronic psychological stress has therefore been associated with increased kynurenine production and altered metabolite profiles, providing a mechanistic link between stress exposure, depression, and cognitive decline.

The gut microbiota has recently emerged as another important regulator of kynurenine pathway activity. Through its effects on tryptophan availability, intestinal barrier integrity, immune signaling, and microbial metabolite production, the gut microbiota may influence the metabolic fate of tryptophan and the balance of kynurenine metabolites [16,17,67,68]. These microbiota-dependent mechanisms are discussed in detail in Section 4.

Beyond its neurological functions, the kynurenine pathway also contributes to cellular energy metabolism through the synthesis of NAD^+^. Several downstream metabolites ultimately serve as precursors for NAD^+^ biosynthesis, linking tryptophan metabolism to mitochondrial function, ATP production, DNA repair, and cellular resilience [58]. Consequently, disturbances in kynurenine metabolism may affect not only neurotransmission and immune regulation but also fundamental processes involved in aging and cellular survival.

Dysregulation of kynurenine metabolism has been implicated in several neuropsychiatric and neurodegenerative disorders, including major depressive disorder and AD. Alterations in kynurenine pathway metabolites have been detected in both peripheral circulation and cerebrospinal fluid, supporting their potential relevance as biomarkers and therapeutic targets [88].

Overall, the kynurenine pathway represents a highly regulated metabolic network linking tryptophan metabolism with immune, neuroendocrine, and CNS function. Its biological effects depend on pathway activity and the relative production of downstream metabolites, providing the biochemical framework for the interactions discussed in the following sections.

**Figure 2:**
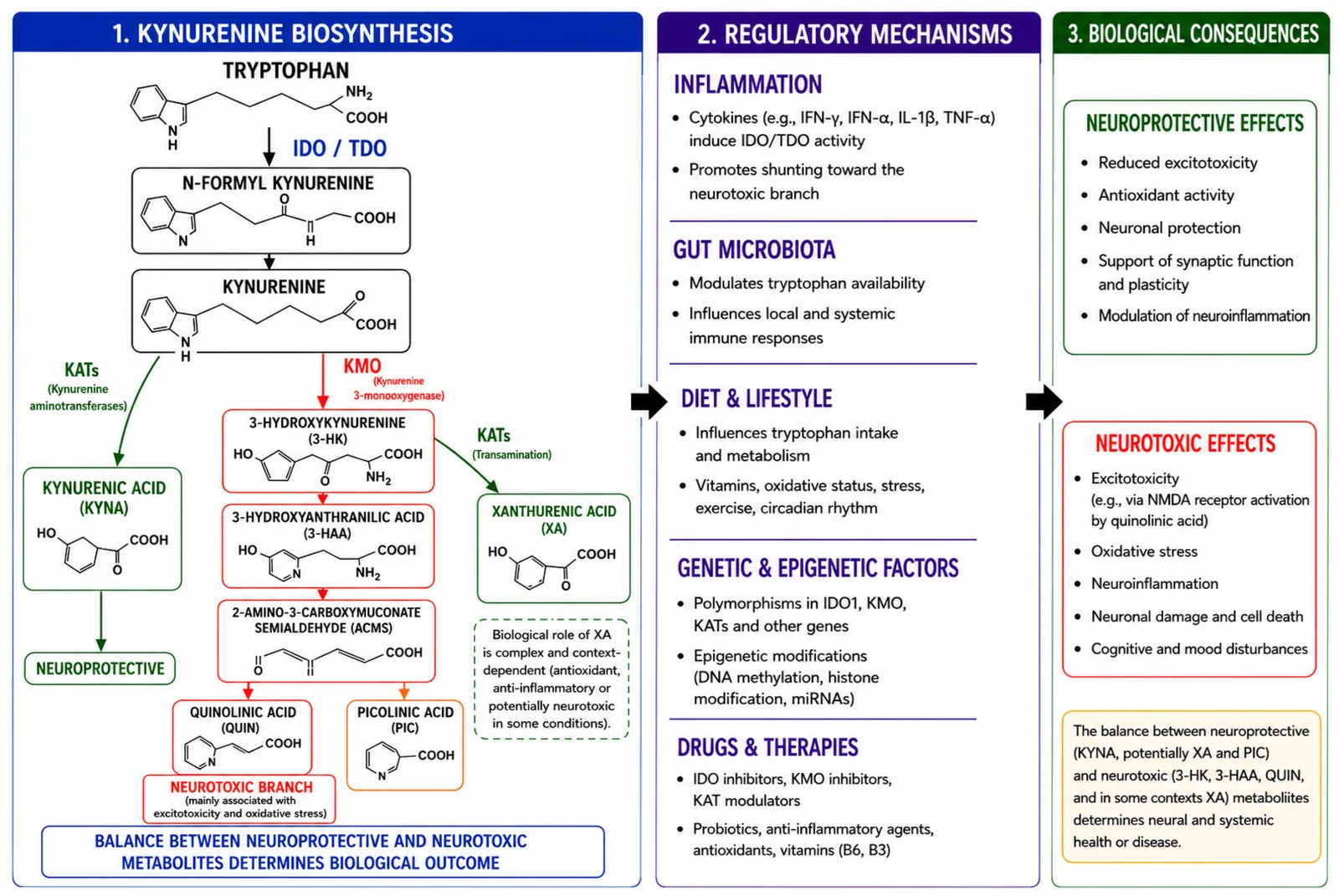
**Kynurenine pathway biosynthesis, regulatory mechanisms, and biological consequences.** Tryptophan is converted to N-formylkynurenine by IDO or TDO and subsequently metabolized to kynurenine. Kynurenine can be directed toward the neuroprotective branch through KATs, leading to the formation of KYNA, or toward the KMO-dependent branch, generating 3-HK and subsequently 3-HAA. Further metabolism of 3-HAA produces ACMS, a metabolic branch-point intermediate that can undergo spontaneous cyclization to form QUIN or be diverted toward the formation of PIC. Kynurenine may also be transaminated to XA, whose biological effects are context-dependent. Kynurenine pathway activity is modulated by inflammation, gut microbiota, diet and lifestyle, genetic and epigenetic factors, and pharmacological interventions. The balance among neuroprotective, neurotoxic, and context-dependent metabolites influences excitotoxicity, oxidative stress, neuroinflammation, neuronal survival, synaptic function, and cognitive and mood-related processes. **Abbreviations:** 3-HAA, 3-hydroxyanthranilic acid; 3-HK, 3-hydroxykynurenine; ACMS, 2-amino-3-carboxymuconate semialdehyde; IDO, indoleamine 2,3-dioxygenase; KATs, kynurenine aminotransferases; KMO, kynurenine 3-monooxygenase; KYNA, kynurenic acid; PIC, picolinic acid; QUIN, quinolinic acid; TDO, tryptophan 2,3-dioxygenase; XA, xanthurenic acid.

The major biochemical steps, regulatory influences, and downstream consequences of kynurenine metabolism are summarized in Figure 2.

### 4.4. Key Enzymes Involved in Kynurenine Metabolism

The kynurenine pathway is regulated by several key enzymes that determine both the rate of tryptophan degradation and the balance between neuroprotective and neurotoxic metabolite production. Among these enzymes, IDO, TDO, KMO, and KATs play particularly important roles in controlling pathway activity and influencing neurological health [17,51].

#### 4.4.1. Indoleamine 2,3-Dioxygenases (IDO1 and IDO2)

Indoleamine 2,3-dioxygenase exists as two distinct isoforms, IDO1 and IDO2, which differ in their expression patterns, regulation, and catalytic properties. IDO1 is the better-characterized isoform and represents a major inflammation-responsive enzyme initiating extrahepatic tryptophan degradation through the kynurenine pathway. In particular, IDO1 expression is strongly induced by pro-inflammatory signals, especially IFN-γ, thereby linking immune activation to increased kynurenine production. IDO2 is a distinct, related enzyme with different tissue distribution and substantially different catalytic characteristics, and its physiological contribution to tryptophan metabolism remains less clearly established. Importantly, the biochemical properties of IDO also show species-dependent differences. Comparative studies of recombinant human and murine IDO have demonstrated differences in substrate conversion, pH stability, inhibitor sensitivity, and thermal stability, indicating that findings obtained in rodent experimental models should be extrapolated to human kynurenine metabolism with appropriate caution [89].

The distinction between IDO isoforms may also be relevant to neuropsychiatric disorders, in which pathway alterations should not necessarily be interpreted as uniform changes in “IDO” activity. Recent evidence in schizophrenia has identified sex-specific associations involving the IDO1 gene together with altered inflammatory cytokines and neuroactive kynurenine metabolites [90]. Although these findings cannot be directly extrapolated to depression or AD, they support the broader concept that kynurenine pathway dysregulation may involve selective, context-dependent alterations of individual enzymes. Accordingly, throughout this review, IDO1 is specified when the available evidence refers specifically to the inflammation-responsive isoform, whereas the broader designation IDO is retained when the original studies did not distinguish between IDO1 and IDO2.

IDO is considered a major molecular link between inflammation and altered neurotransmitter metabolism. During chronic inflammatory states, increased IDO activity accelerates tryptophan degradation, reducing its availability for serotonin synthesis while increasing kynurenine production. This metabolic shift contributes to decreased serotonergic neurotransmission and enhanced generation of downstream neuroactive metabolites [5,58,86].

#### 4.4.2. Tryptophan 2,3-Dioxygenase (TDO)

TDO catalyzes the same initial reaction as IDO but differs substantially in its tissue distribution and regulatory mechanisms. TDO is predominantly expressed in the liver and serves as the principal regulator of systemic tryptophan homeostasis under physiological conditions [64]. The activity of TDO is stimulated by elevated circulating tryptophan concentrations and by glucocorticoids released during activation of the HPA axis [87].

Through its sensitivity to stress hormones, TDO provides a direct biochemical connection between chronic stress and kynurenine metabolism. Persistent activation of the HPA axis leads to increased cortisol secretion, which enhances TDO activity and promotes kynurenine production. This mechanism may contribute to reduced serotonin availability and altered neurochemical balance during prolonged stress exposure [51,82].

The relative contribution of IDO and TDO to kynurenine pathway dysregulation may differ according to disease context and tissue compartment. In MDD, peripheral immune activation may enhance IDO activity in circulating and tissue-resident immune cells, thereby increasing systemic tryptophan degradation and kynurenine production [88]. In parallel, chronic activation of the hypothalamic–pituitary–adrenal axis may stimulate predominantly hepatic TDO through glucocorticoid signaling, providing a complementary route by which psychological or physiological stress can alter systemic tryptophan availability [64,66]. Because kynurenine can cross the blood–brain barrier, peripheral kynurenine production may influence central kynurenine metabolism [65]. Within the CNS, locally expressed IDO, particularly in activated microglia and other inflammatory cells, provides an additional mechanism linking neuroinflammation to altered kynurenine metabolism [87,88]. In AD, where chronic neuroinflammation and microglial activation are prominent pathological features, inflammation-responsive IDO activity within the CNS may therefore be particularly relevant, while peripheral kynurenine metabolism may additionally contribute to the substrate available for cerebral kynurenine pathway activity [88]. TDO should not, however, be regarded as exclusively peripheral, because although its activity is predominantly hepatic, TDO expression is also present within the brain [65,87]. Thus, rather than representing disease-specific enzymes, IDO and TDO constitute partially overlapping regulatory routes whose relative contribution depends on inflammatory status, HPA-axis activity, tissue localization, and disease stage.

#### 4.4.3. Kynurenine Monooxygenase (KMO)

KMO is a pivotal enzyme that directs kynurenine metabolism toward the neurotoxic branch of the pathway. KMO converts kynurenine into 3-hydroxykynurenine, a metabolite that subsequently gives rise to 3-hydroxyanthranilic acid and quinolinic acid [89,90]. KMO is highly expressed in activated microglia, macrophages, and peripheral immune cells and is often upregulated during inflammatory conditions.

Because KMO controls the formation of several potentially neurotoxic metabolites, it is considered one of the most important determinants of kynurenine pathway-related neurodegeneration. By directing kynurenine toward 3-HK and QUIN formation, increased KMO activity can favor the production of metabolites associated with oxidative and excitotoxic effects [82].

#### 4.4.4. Kynurenine Aminotransferases (KATs)

Kynurenine aminotransferases (KATs) comprise a family of four mammalian enzymes, commonly designated KAT I–IV, which catalyze the transamination of kynurenine to KYNA. Although these enzymes share the capacity to generate KYNA, they differ in their biochemical properties, substrate preferences, and cellular and tissue distribution. Consequently, alterations in KYNA concentrations should not necessarily be interpreted as reflecting uniform changes in total KAT activity because individual KAT isoforms may contribute differently to KYNA synthesis and may be differentially regulated under physiological and pathological conditions [91,92].

This distinction may be particularly relevant under chronic stress, an important risk factor for depression. Experimental evidence indicates that chronic mild stress can alter kynurenine pathway metabolism in the frontal cortex and modify glutamatergic neurotransmission [93]. These findings support the importance of considering enzyme- and isoform-specific regulation when interpreting stress-related alterations in kynurenine metabolism and KYNA-related signaling.

KYNA exerts several beneficial effects within the brain. It functions as an endogenous antagonist of NMDA receptors and modulates α7 nicotinic acetylcholine receptors, thereby limiting excessive glutamatergic neurotransmission and protecting neurons against excitotoxic injury [88]. In addition, KYNA possesses antioxidant and anti-inflammatory properties that may contribute to neuronal survival under conditions of oxidative stress and chronic inflammation. The balance between KMO-mediated neurotoxic metabolite production and KAT-mediated KYNA synthesis is therefore considered a key determinant of neurological outcomes. Disruption of this balance may favor neurotoxic metabolite production and impair neuronal homeostasis [17,51].

Taken together, IDO, TDO, KMO, and KATs regulate the major branching points of the kynurenine pathway and determine the metabolic fate of tryptophan. Their coordinated activity influences the balance between serotonin synthesis, neuroprotective metabolite formation, and neurotoxic metabolite accumulation. Dysregulation of these enzymes may therefore contribute to neuroinflammation, oxidative stress, impaired neurotransmission, and neurodegeneration, highlighting their importance within the microbiota–gut–brain axis and their relevance to both depression and AD.

### 4.5. Neuroactive Metabolites of the Kynurenine Pathway

The biological significance of the kynurenine pathway largely depends on the production of several neuroactive metabolites capable of influencing neurotransmission, oxidative balance, neuroinflammation, and neuronal survival. These metabolites exert diverse and often opposing effects within the CNS, ranging from neuroprotection to neurotoxicity. The balance between their production is considered a critical determinant of brain homeostasis and neurological health. Among the most extensively studied kynurenine metabolites are KYNA, QUIN, 3-HK, and anthranilic acid, each contributing differently to the pathophysiology of depression, cognitive decline, and neurodegenerative disorders [51,83].

#### 4.5.1. Kynurenic Acid (KYNA)

KYNA is a major neuroactive metabolite of the kynurenine pathway and is primarily synthesized from kynurenine through the action of KATs, particularly within astrocytes [89]. Under physiological conditions, KYNA contributes to the regulation of excitatory neurotransmission and neuronal homeostasis. Its neuroprotective properties are mainly attributed to its ability to limit excessive glutamatergic signaling through antagonism of NMDA receptors and modulation of α7 nicotinic acetylcholine receptor-mediated neurotransmission [93]. By reducing excessive excitatory signaling, KYNA may protect neurons from excitotoxic injury. However, its biological effects are not uniformly beneficial and depend on concentration and neurobiological context. Excessive KYNA may interfere with physiological glutamatergic and cholinergic neurotransmission and thereby impair synaptic plasticity and cognitive function. This dual role is particularly relevant to schizophrenia, in which increased KYNA has been proposed to contribute to glutamatergic hypofunction and cognitive disturbances [94]. Thus, rather than being considered intrinsically neuroprotective, KYNA should be regarded as a context-dependent neuroactive metabolite whose effects depend on its concentration, receptor interactions, and the underlying pathological state. Consequently, maintaining an appropriate balance between KYNA and other kynurenine pathway metabolites appears important for neurological homeostasis [93,94].

#### 4.5.2. Quinolinic Acid (QUIN)

QUIN is one of the most important neurotoxic metabolites generated within the kynurenine pathway. It is produced predominantly by activated microglia and infiltrating macrophages through the downstream metabolism of 3-HK and 3-hydroxyanthranilic acid [87]. Under physiological conditions, QUIN serves as an intermediate in NAD^+^ biosynthesis; however, excessive accumulation may exert detrimental neurological effects. The neurotoxicity of QUIN is largely mediated through its role as an endogenous agonist of NMDA receptors. Excessive NMDA receptor activation results in increased intracellular calcium concentrations, activation of proteolytic enzymes, mitochondrial dysfunction, and generation of ROS [17]. These events ultimately promote neuronal damage and apoptosis. In addition to excitotoxicity, QUIN contributes to neuroinflammation by stimulating microglial activation and promoting the release of pro-inflammatory cytokines. Elevated QUIN concentrations have been reported in several neuropsychiatric and neurodegenerative disorders [86]. In AD, QUIN has been associated with amyloid-β accumulation, tau hyperphosphorylation, synaptic dysfunction, and progressive neuronal loss [88].

#### 4.5.3. 3-Hydroxykynurenine (3-HK)

3-HK occupies a central position within the neurotoxic branch of the kynurenine pathway and is generated through the action of KMO [95]. Unlike QUIN, which exerts its effects primarily through excitotoxic mechanisms, the biological activity of 3-HK is mainly related to oxidative stress. The molecule readily undergoes auto-oxidation, leading to the formation of ROS, including superoxide anions and hydrogen peroxide [84]. Excessive production of these reactive molecules can damage cellular proteins, membrane lipids, and nucleic acids, ultimately impairing neuronal viability. Furthermore, 3-HK may reduce intracellular antioxidant defenses and exacerbate mitochondrial dysfunction, thereby amplifying oxidative injury [96]. Because oxidative stress is recognized as a major contributor to both depression and neurodegeneration, increased 3-HK concentrations are considered an important pathogenic factor linking inflammation to neuronal damage. Increased 3-HK concentrations have been associated with oxidative stress-related neurological disorders [97].

#### 4.5.4. Anthranilic Acid

Anthranilic acid is a lesser-studied kynurenine metabolite generated from kynurenine by kynureninase [58]. Emerging evidence suggests potential antioxidant and immunomodulatory properties, although its precise biological role remains incompletely understood [15,66,98]. The neuroactive metabolites of the kynurenine pathway exert diverse and often opposing effects on brain physiology. While KYNA generally promotes neuroprotection and limits excitotoxicity, QUIN and 3-HK contribute to neuroinflammation, oxidative stress, and neuronal injury. Anthranilic acid may represent an additional regulatory metabolite with potential immunomodulatory functions. The balance among these metabolites is therefore considered a crucial determinant of neurological health and a key factor linking inflammation, gut dysbiosis, depression, and AD [51,83].

#### 4.5.5. Picolinic Acid (PIC)

PIC is another downstream metabolite of the kynurenine pathway. It is generated from 2-amino-3-carboxymuconate semialdehyde (ACMS) through an alternative metabolic route that competes with the spontaneous formation of QUIN, thereby influencing the distribution of downstream kynurenine metabolites. PIC has been associated with metal-chelating and immunomodulatory properties and may contribute to the regulation of kynurenine pathway homeostasis [17].

#### 4.5.6. 3-Hydroxyanthranilic Acid (3-HAA)

3-HAA is a downstream metabolite of the kynurenine pathway formed from 3-HK through the action of kynureninase. It is subsequently converted by 3-hydroxyanthranilate 3,4-dioxygenase (3-HAO) into ACMS, an important branch-point intermediate leading toward quinolinic acid and NAD^+^ biosynthesis. 3-HAA exhibits complex redox and immunomodulatory properties and, under specific conditions, may contribute to oxidative stress [17,84].

## 5. Interactions Between Gut Microbiota and the Tryptophan–Kynurenine Pathway

### 5.1. Microbial Regulation of Tryptophan Availability

Tryptophan availability represents a critical factor determining the balance between the major metabolic pathways of tryptophan degradation, including serotonin synthesis, kynurenine pathway activation, and microbial indole production. As an essential amino acid, tryptophan cannot be synthesized by humans and must be obtained through dietary intake. However, the amount of tryptophan that ultimately becomes available to host tissues is not determined solely by nutritional intake but is also strongly influenced by the gut microbiota. Through its effects on nutrient metabolism, intestinal absorption, immune signaling, and microbial competition, the gut microbiota plays a central role in regulating systemic and central tryptophan availability [14,67,81].

One of the primary mechanisms through which gut microorganisms regulate tryptophan availability involves direct competition with the host for dietary tryptophan. Many intestinal bacterial species require tryptophan for their own growth and metabolic activities. Consequently, a portion of dietary tryptophan is utilized by microbial communities before it can be absorbed by the host [68]. Under physiological conditions, this competition contributes to metabolic homeostasis and supports the production of beneficial microbial metabolites. However, alterations in microbial composition may substantially modify the amount of tryptophan available for host metabolism.

Certain bacterial taxa possess specialized enzymatic systems capable of converting tryptophan into a wide range of bioactive compounds. Species belonging to the genera *Lactobacillus*, *Bifidobacterium*, *Clostridium*, *Peptostreptococcus*, and *Bacteroides* metabolize tryptophan into indole derivatives, including indole-3-acetic acid (IAA), indole-3-propionic acid (IPA), indole-3-aldehyde, indoleacrylic acid, and tryptamine [96]. These microbial metabolites are increasingly recognized as important signaling molecules that influence intestinal barrier integrity, immune regulation, and communication within the microbiota–gut–brain axis. An important translational consideration is that microbial tryptophan metabolism depends strongly on the taxonomic and functional composition of the gut microbiota, which differs between rodents and humans. Bacterial taxa vary considerably in their enzymatic capacity to metabolize tryptophan and consequently generate distinct profiles of bioactive metabolites, including indole, indole-3-lactic acid, indole-3-acetic acid, indole-3-propionic acid, indole-3-aldehyde, and tryptamine [89,90,99]. Therefore, differences in microbial community composition between experimental animals and humans may result in distinct tryptophan-derived metabolite profiles and potentially different downstream effects on intestinal barrier function, immune signaling, AhR activation, and host tryptophan metabolism. Consequently, findings obtained in rodent models should not be assumed to reproduce the human microbiota–tryptophan axis directly, and species-specific microbial composition should be considered when translating preclinical findings to human depression and AD [89,90].

The regulation of tryptophan availability extends beyond direct microbial utilization. Gut microbiota also influences the efficiency of intestinal absorption. The intestinal epithelium expresses several amino acid transport systems responsible for transferring tryptophan from the intestinal lumen into the circulation. Emerging evidence suggests that microbial metabolites can regulate the expression and activity of these transporters, thereby affecting host tryptophan uptake [100]. In addition, microbial modulation of epithelial integrity influences the absorptive capacity of the intestinal mucosa and may indirectly alter amino acid bioavailability. After absorption, circulating tryptophan competes with other large neutral amino acids for transport across the blood–brain barrier, thereby influencing central tryptophan availability [60].

Gut microbiota also influences tryptophan availability indirectly through immune regulation. Dysbiosis-associated increases in pro-inflammatory cytokines can stimulate IDO activity, thereby accelerating tryptophan degradation and favoring kynurenine formation at the expense of serotonin synthesis [16,66,101]. This immunometabolic mechanism is discussed in greater detail in Section 5.3 and Section 5.4.

Recent studies have also demonstrated that microbial metabolites can influence host tryptophan metabolism through activation of the aryl hydrocarbon receptor (AhR), a ligand-activated transcription factor expressed in epithelial cells, immune cells, and neurons [102]. Several microbiota-derived indole compounds act as endogenous AhR ligands and regulate genes involved in immune responses, epithelial barrier maintenance, and intestinal homeostasis. Activation of AhR signaling promotes mucosal integrity and suppresses excessive inflammation, thereby indirectly preserving physiological tryptophan metabolism [103].

Current evidence demonstrates that gut microbiota regulate tryptophan availability through multiple complementary mechanisms, including direct microbial utilization of tryptophan, production of microbial metabolites, modulation of intestinal absorption, regulation of immune responses, and control of kynurenine pathway activation. Through these interconnected processes, intestinal microorganisms determine the amount of tryptophan available for serotonin synthesis, kynurenine metabolism, and microbial biotransformation. Disturbances in microbial composition may significantly alter tryptophan homeostasis and contribute to the pathogenesis of depression, cognitive decline, and neurodegenerative disorders.

### 5.2. Gut Microbiota-Derived Metabolites and Host Metabolism

Beyond regulating tryptophan availability, the gut microbiota exerts profound effects on host physiology through the production of a diverse array of bioactive metabolites. These microbial-derived compounds function as signaling molecules that influence intestinal homeostasis, immune responses, endocrine regulation, energy metabolism, and brain function. Increasing evidence suggests that microbial metabolites constitute one of the principal mechanisms through which the gut microbiota communicates with distant organs, including the CNS, thereby playing a fundamental role within the microbiota–gut–brain axis [67,104].

Among the most extensively studied microbial metabolites are SCFAs, including acetate, propionate, and butyrate. These metabolites contribute to barrier integrity, immune regulation, metabolic homeostasis, and gut–brain communication [36,55,56,88,105,106,107].

Another important class of microbial metabolites derives directly from tryptophan metabolism. Various intestinal microorganisms convert dietary tryptophan into indole and a variety of indole derivatives, including IAA, IPA, indole-3-aldehyde, indoleacrylic acid, and tryptamine [68]. These compounds exert important biological effects through activation of the AhR, a ligand-activated transcription factor involved in immune regulation and epithelial homeostasis.

Activation of AhR signaling by microbial indole derivatives promotes epithelial barrier integrity, enhances mucosal immune tolerance, and suppresses excessive inflammatory responses [102]. In the intestinal environment, AhR activation stimulates the production of interleukin-22 (IL-22), which promotes epithelial regeneration, antimicrobial peptide synthesis, and maintenance of mucosal defense mechanisms [108]. Consequently, microbial indole metabolites contribute significantly to intestinal homeostasis and may indirectly influence systemic inflammation and brain health.

IPA exhibits antioxidant and neuroprotective properties and may contribute to the maintenance of blood–brain barrier integrity [109,110]. Tryptamine is a microbial tryptophan metabolite that may influence serotonergic signaling and gut–brain communication [111].

Gut microorganisms also transform primary bile acids into secondary bile acids that regulate metabolic, immune, and neuroendocrine pathways through receptors such as Farnesoid X Receptor (FXR) and Takeda G protein-coupled receptor 5 (TGR5) [112,113,114].

Gut microbiota also contributes to host metabolism through the production of vitamins and cofactors involved in neuronal function. Various bacterial species synthesize B vitamins, including folate, riboflavin, pyridoxine, and cobalamin, which participate in neurotransmitter synthesis, methylation reactions, and energy metabolism [115]. Alterations in microbial vitamin production may therefore influence neurochemical homeostasis and cognitive function.

Importantly, microbial metabolites interact extensively with host immune and metabolic pathways. Dysbiosis-induced alterations in metabolite production may shift the balance from anti-inflammatory and neuroprotective signaling toward a pro-inflammatory state characterized by impaired barrier function, immune activation, and altered neurotransmitter metabolism [10].

Gut microbiota-derived metabolites constitute a major communication system linking the intestinal microbiome to host physiology. Through the production of SCFAs, indole derivatives, bile acid metabolites, vitamins, and other signaling molecules, intestinal microorganisms regulate metabolic homeostasis, immune responses, epithelial barrier function, and CNS activity. Disturbances in microbial metabolite production may therefore represent a critical mechanistic link between gut dysbiosis, altered tryptophan metabolism, neuroinflammation, and the development of depression and AD.

### 5.3. Dysbiosis and Alterations in Kynurenine Pathway Activity

Gut microbial homeostasis is essential for maintaining physiological tryptophan metabolism and balanced kynurenine pathway activity. Under healthy conditions, interactions between commensal microorganisms, intestinal epithelial cells, and the immune system help preserve metabolic equilibrium and regulate the partitioning of tryptophan between serotonin synthesis, microbial metabolism, and kynurenine production. However, alterations in microbial composition and diversity, collectively referred to as dysbiosis, may profoundly disrupt these regulatory mechanisms and contribute to abnormal activation of the kynurenine pathway [14,67].

Dysbiosis is characterized by a reduction in microbial diversity, loss of beneficial commensal species, and expansion of potentially pathogenic microorganisms. Multiple factors may contribute to its development, including aging, chronic stress, Western dietary patterns, infections, antibiotic exposure, sedentary lifestyle, and chronic diseases [10]. These microbial alterations affect both local intestinal physiology and systemic metabolic pathways, creating conditions that favor inflammation and altered tryptophan metabolism.

One of the most important consequences of dysbiosis is the disruption of immune homeostasis. Beneficial bacterial taxa normally promote regulatory immune responses and suppress excessive inflammation through the production of SCFAs, indole derivatives, and other immunomodulatory metabolites [105]. When these microorganisms decline, anti-inflammatory signaling decreases, whereas pro-inflammatory pathways become increasingly activated. As a result, elevated production of cytokines such as IFN-γ, TNF-α, IL-1β, and IL-6 is frequently observed [49].

These inflammatory mediators play a pivotal role in regulating kynurenine pathway activity. IFN-γ is considered the most potent inducer of IDO, the rate-limiting enzyme responsible for converting tryptophan into kynurenine [66]. Activation of IDO accelerates tryptophan degradation and diverts metabolism away from serotonin synthesis toward kynurenine production. Consequently, chronic inflammation associated with dysbiosis leads to decreased serotonin availability and increased generation of downstream kynurenine metabolites [16].

Increased peripheral kynurenine production may influence brain kynurenine metabolism because kynurenine readily crosses the blood–brain barrier [17].

Importantly, inflammatory conditions appear to preferentially activate the neurotoxic branch of the kynurenine pathway. Under inflammatory conditions, kynurenine metabolism shifts toward the production of neurotoxic metabolites such as 3-HK and QUIN [82]. Aging itself further amplifies these processes. Age-related reductions in microbial diversity are commonly accompanied by increased intestinal permeability, chronic low-grade inflammation, and persistent activation of the kynurenine pathway [116].

Recent advances in metagenomic and metabolomic technologies have provided additional evidence linking microbial composition to kynurenine pathway activity. Specific microbial taxa, including members of the genera *Faecalibacterium*, *Roseburia*, *Bifidobacterium*, and *Lactobacillus*, have been associated with lower inflammatory activity and more favorable kynurenine metabolite profiles [117]. Conversely, increased abundance of pro-inflammatory taxa has been correlated with elevated kynurenine concentrations and enhanced neurotoxic metabolite production [118].

Current evidence indicates that gut dysbiosis profoundly influences kynurenine pathway activity through mechanisms involving immune activation, cytokine production, altered tryptophan availability, and modulation of key metabolic enzymes. These alterations shift tryptophan metabolism toward increased production of neuroactive kynurenine metabolites and may represent a central mechanism linking gut microbial disturbances to depression, cognitive decline, and AD.

### 5.4. Intestinal Permeability, Inflammation, and Immune Activation

The intestinal barrier represents one of the most important interfaces between the external environment and the host. Under physiological conditions, this highly specialized structure allows the selective absorption of nutrients while preventing the translocation of pathogens, toxins, and microbial products into the systemic circulation. Maintenance of intestinal barrier integrity is therefore essential for preserving metabolic homeostasis, immune tolerance, and normal gut–brain communication [119,120].

The intestinal barrier consists of several interconnected components, including the mucus layer, intestinal epithelial cells, tight junction proteins, antimicrobial peptides, immune cells, and the gut microbiota itself. Tight junction proteins such as occludin, claudins, and ZO-1 form a highly regulated seal between adjacent epithelial cells and play a crucial role in controlling paracellular permeability [121]. Together, these structures create a dynamic barrier that continuously adapts to dietary, microbial, and environmental stimuli.

Gut microbiota contribute significantly to the maintenance of barrier integrity. Beneficial microorganisms stimulate mucus production, enhance epithelial renewal, regulate tight junction expression, and produce metabolites such as SCFAs that support epithelial health [105]. Butyrate, in particular, serves as the primary energy source for colonocytes and promotes the expression of tight junction proteins while suppressing inflammatory signaling pathways [55]. Through these mechanisms, a balanced microbiota helps preserve intestinal homeostasis and limits systemic exposure to potentially harmful microbial components.

When dysbiosis develops, however, intestinal barrier function may become compromised. Reductions in beneficial bacterial populations, combined with expansion of pro-inflammatory microorganisms, can impair mucus production, disrupt tight junction organization, and weaken epithelial integrity [104]. This condition, commonly referred to as increased intestinal permeability or “leaky gut,” allows luminal antigens and microbial-derived molecules to cross the intestinal barrier and enter the circulation.

Among the microbial products capable of translocating across a compromised intestinal barrier, LPS has attracted particular attention. LPS is a major component of the outer membrane of Gram-negative bacteria and acts as a potent activator of innate immune responses [122]. Once present in the bloodstream, LPS binds to TLR4 expressed on macrophages, dendritic cells, endothelial cells, and other immune cells. Activation of TLR4 initiates intracellular signaling cascades involving NF-κB, leading to the production of numerous pro-inflammatory mediators [123].

As a consequence, increased circulating concentrations of cytokines such as TNF-α, IL-1β, IL-6, and IFN-γ are frequently observed in individuals with intestinal barrier dysfunction [49]. These cytokines exert widespread systemic effects and serve as critical messengers linking peripheral immune activation to CNS dysfunction.

Peripheral cytokines can influence the brain through multiple routes, including blood–brain barrier signaling and activation of neuroimmune pathways [124].

Chronic inflammatory stimulation promotes microglial activation and neuroinflammation, contributing to neuronal dysfunction and neurodegeneration [125]. Pro-inflammatory cytokines activate IDO and promote kynurenine pathway activation, increasing the generation of neuroactive metabolites with potential neurotoxic effects [17,66].

Aging further amplifies these processes through progressive loss of microbial diversity and development of chronic low-grade inflammation [116].

The available evidence indicates that intestinal permeability represents a crucial mechanistic link between gut dysbiosis and neurological disease. Disruption of epithelial barrier integrity facilitates the translocation of microbial products that trigger systemic immune activation, chronic inflammation, and kynurenine pathway dysregulation. Through these interconnected mechanisms, intestinal barrier dysfunction may contribute significantly to the pathogenesis of depression, cognitive decline, and AD. An important consideration when interpreting these mechanisms is the distinction between evidence obtained from experimental models and that derived from human studies. Much of the detailed mechanistic evidence linking dysbiosis, disruption of epithelial tight junctions, microbial product translocation, TLR4/NF-κB signaling, systemic cytokine production, and subsequent neuroimmune alterations has been established in cellular and animal models. Human studies provide complementary evidence for associations among altered gut microbiota composition, markers of intestinal permeability, systemic inflammation, and neuropsychiatric or neurodegenerative disorders; however, these studies do not always establish the same causal sequence demonstrated experimentally [11,26]. Moreover, differences between experimental animals and humans in microbiota composition, host physiology, immune responses, and environmental exposures may substantially influence microbiota–host interactions and limit the direct extrapolation of preclinical findings to human disease [11,26]. Accordingly, mechanistic findings from rodent models should be interpreted as providing biological plausibility rather than direct evidence that identical pathways operate with the same magnitude or temporal sequence in humans. Future translational studies integrating microbiome profiles, intestinal permeability markers, inflammatory mediators, and kynurenine pathway metabolites in well-characterized human cohorts will be necessary to establish the clinical relevance of these mechanistic links.

**Figure 3:**
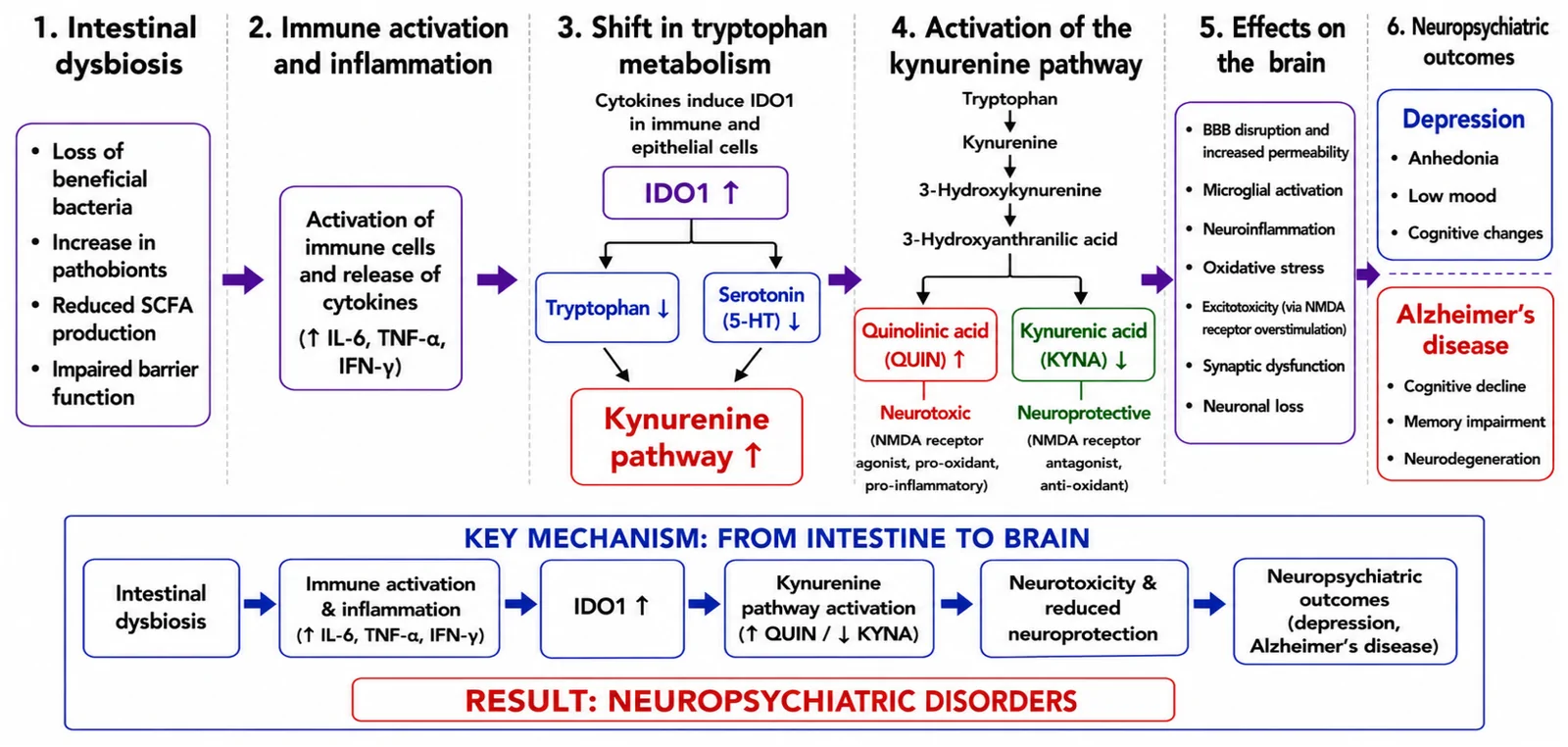
Proposed integrative microbiota–immune–tryptophan model linking intestinal dysbiosis to depression and Alzheimer’s disease. The model proposed in this review positions the kynurenine pathway as a dynamic molecular interface connecting intestinal dysbiosis, immune activation, altered tryptophan metabolism, and central nervous system dysfunction. Intestinal dysbiosis promotes immune activation and the release of pro-inflammatory cytokines, including IL-6, TNF-α, and IFN-γ, which stimulate IDO1 activity and alter tryptophan metabolism. Increased diversion of tryptophan toward the kynurenine pathway may reduce its availability for serotonin synthesis and favor an imbalance between neurotoxic and neuroprotective kynurenine metabolites. Increased QUIN relative to KYNA may promote excitotoxicity, oxidative stress, microglial activation, neuroinflammation, synaptic dysfunction, and neuronal injury. Collectively, these interconnected mechanisms provide a potential microbiota–immune–metabolic pathway linking intestinal dysbiosis with neuropsychiatric and neurodegenerative outcomes, including depression and AD. **Abbreviations:** BBB, blood–brain barrier; IDO1, indoleamine 2,3-dioxygenase 1; IFN-γ, interferon-γ; IL-6, interleukin-6; KYNA, kynurenic acid; QUIN, quinolinic acid; SCFAs, short-chain fatty acids; TNF-α, tumor necrosis factor-α; 5-HT, serotonin (5-hydroxytryptamine).

The interconnected mechanisms through which gut dysbiosis influences tryptophan metabolism and kynurenine pathway activity, ultimately contributing to neuropsychiatric and neurodegenerative alterations, are summarized in Figure 3.

## 6. The Tryptophan–Kynurenine Pathway in Depression

### 6.1. Neuroinflammation and Depression

Major depressive disorder is increasingly understood as a heterogeneous condition in which immune dysregulation and neuroinflammation play an important role, particularly in patients with treatment-resistant symptoms, fatigue, anhedonia, cognitive impairment, or comorbid inflammatory diseases [126,127]. Although classical models emphasized monoamine deficiency, current evidence indicates that inflammatory processes can alter neurotransmitter synthesis, neuroendocrine regulation, synaptic plasticity, glutamatergic signaling, and neuronal survival, all of which are relevant to depressive symptomatology [128].

Numerous studies have reported elevated concentrations of IL-6, TNF-α, IL-1β, IFN-γ, and C-reactive protein in patients with major depressive disorder, supporting a role for chronic low-grade inflammation in disease pathogenesis [16,126,127].

Peripheral cytokines can activate neuroimmune pathways and promote microglial activation, contributing to impaired neuroplasticity, synaptic dysfunction, and altered emotional regulation [129,130].

One of the most important biochemical consequences of neuroinflammation in depression is the activation of the tryptophan–kynurenine pathway. This pathway links immune activation to neurotransmitter imbalance because inflammatory cytokines redirect tryptophan metabolism away from serotonin synthesis and toward kynurenine production [131]. In this context, depression may result not only from reduced monoaminergic signaling but also from the accumulation of neuroactive kynurenine metabolites capable of influencing glutamatergic neurotransmission, oxidative stress, and neuronal integrity [16].

### 6.2. Cytokine-Induced Activation of Indoleamine 2,3-Dioxygenase (IDO)

As discussed in Section 4.4.1, two IDO isoforms, IDO1 and IDO2, have been identified. However, much of the literature investigating inflammation-associated kynurenine pathway activation in depression has used the broader designation “IDO” without consistently distinguishing between the two isoforms. Therefore, the term IDO is retained in this section when referring to studies in which isoform-specific involvement was not established. Where specifically demonstrated, IDO1 is identified accordingly.

IDO is a key enzyme connecting inflammation to tryptophan metabolism. IDO catalyzes the initial and rate-limiting conversion of tryptophan into N-formylkynurenine, which is subsequently converted into kynurenine [66]. Although IDO contributes to immune regulation under physiological conditions, persistent activation during chronic inflammation may become maladaptive. The strongest inducer of IDO is IFN-γ, although TNF-α, IL-1β, and IL-6 can further enhance its expression. Mechanistically, IFN-γ activates the JANUS kinase/Signal Transducer and Activator of Transcription 1 (JAK/STAT1) signaling pathway, leading to transcriptional upregulation of the IDO gene. TLR activation by microbial products, such as LPS, may further amplify this response through NF-κB-dependent inflammatory signaling [132]. As a result, inflammatory states increase the kynurenine-to-tryptophan ratio, which is frequently used as an indirect marker of IDO activity [133].

In depression, this mechanism is highly relevant because chronic low-grade inflammation can maintain sustained activation of IDO. The consequence is a metabolic shift in which tryptophan is increasingly degraded through the kynurenine pathway rather than being used for serotonin synthesis. This process provides a direct biochemical explanation for how immune activation may generate depressive symptoms, particularly in patients with elevated inflammatory biomarkers [134].

The distinction between IDO isoforms may nevertheless be important in psychiatric disorders. Recent evidence from schizophrenia has identified sex-specific associations involving the IDO1 gene together with alterations in inflammatory cytokines and neuroactive kynurenine metabolites [89]. Although these findings cannot be directly extrapolated to MDD, they highlight the need for future studies of depression to distinguish IDO1 from IDO2 rather than interpreting changes in kynurenine metabolism as reflecting uniform “IDO” activity.

### 6.3. Reduced Serotonin Synthesis and Depressive Symptoms

Tryptophan is the essential precursor of serotonin, a neurotransmitter involved in mood regulation, sleep, appetite, emotional behavior, and cognitive function. During inflammatory activation, increased IDO activity promotes tryptophan catabolism through the kynurenine pathway, potentially reducing tryptophan availability for serotonin biosynthesis [135]. This does not mean that depression is caused only by serotonin deficiency, but rather that reduced serotonin availability may represent one component of a broader inflammatory-metabolic disturbance.

Reduced serotonin availability may contribute to depressive symptoms, including low mood, anhedonia, sleep disturbances, and impaired cognitive flexibility [64,77].

Importantly, the same inflammatory state that decreases serotonin availability also increases kynurenine production. Thus, depressive symptoms may arise from a dual mechanism: reduced serotonin-mediated neurotransmission and increased production of kynurenine metabolites that affect glutamatergic signaling, oxidative stress, and neuroinflammation [17]. This model helps explain why some patients with depression, especially those with elevated C-reactive protein or cytokine levels, respond poorly to conventional antidepressants that primarily target monoamine reuptake [126,136].

### 6.4. Neurotoxic Kynurenine Metabolites in Mood Disorders

In the brain, kynurenine may be metabolized into neuroprotective KYNA or neurotoxic metabolites such as 3-HK and QUIN [16,82]. 3-HK promotes oxidative stress and mitochondrial dysfunction, processes implicated in depressive symptomatology [84,137]. QUIN promotes excitotoxicity through NMDA receptor activation and contributes to glutamatergic dysregulation, a mechanism increasingly implicated in severe and treatment-resistant depression [16,83,94]. A shift toward increased QUIN and reduced KYNA favors neuroinflammation, impaired synaptic plasticity, and persistence of depressive symptoms [97].

Importantly, central kynurenine pathway alterations appear to exhibit regional heterogeneity rather than occurring uniformly throughout the brain. Preclinical studies of stress- and inflammation-related depressive phenotypes have demonstrated alterations in kynurenine pathway metabolism in brain regions critically involved in mood, cognition, and stress regulation, including the hippocampus, prefrontal cortex, amygdala, and hypothalamus [138]. Such regional differences may be functionally relevant because the hippocampus and prefrontal cortex are critically involved in cognitive and emotional regulation, whereas the amygdala contributes to affective and stress-related processing. However, much of the evidence describing region-specific kynurenine pathway changes derives from experimental models, and direct evidence demonstrating comparable regional metabolic alterations in the human MDD brain remains more limited.

### 6.5. Clinical Evidence Linking Kynurenine Metabolites to Depression

Clinical studies increasingly support the involvement of kynurenine pathway dysregulation in major depressive disorder. Meta-analyses have reported altered concentrations of tryptophan, kynurenine, KYNA, QUIN, and related metabolite ratios in patients with depression compared with healthy controls. Despite methodological differences between studies, the overall evidence indicates significant alterations in tryptophan metabolism in depression [86,139].

One of the most consistent findings is reduced tryptophan availability and altered kynurenine pathway activity in active depression [23]. Some studies report increased KYN/TRP ratios, suggesting enhanced IDO or TDO activity, particularly in patients with inflammatory phenotypes [85,131]. Other studies show reduced KYNA or altered KYNA to QUIN ratios, suggesting a shift away from neuroprotection and toward neurotoxic metabolite production [140].

Evidence also indicates that kynurenine pathway abnormalities may be linked to symptom profiles. Increased QUIN and 3-HK have been associated with anhedonia, psychomotor slowing, cognitive dysfunction, and suicidal behavior in some studies [97]. These associations are biologically plausible because neurotoxic kynurenine metabolites affect glutamate signaling, oxidative stress, and neuronal plasticity, all of which are involved in emotional and cognitive regulation.

The kynurenine pathway may also have value as a biomarker system. The KYN/TRP ratio may reflect inflammatory activation of tryptophan catabolism, while the KYNA to QUIN ratio may indicate the balance between neuroprotective and neurotoxic pathway activity [83,133]. However, these markers are not yet sufficiently standardized for routine clinical use.

Overall, the tryptophan–kynurenine pathway provides a mechanistic framework linking inflammation, reduced serotonin synthesis, glutamatergic dysregulation, oxidative stress, and impaired neuroplasticity in depression. Its relevance is particularly strong in inflammatory depression, treatment-resistant depression, and depressive states associated with aging, metabolic disease, or gut dysbiosis.

Clinical studies provide consistent evidence that major depressive disorder is associated with alterations in tryptophan–kynurenine metabolism, although the direction and magnitude of these changes vary according to inflammatory status, disease stage, age, sex, and treatment exposure. Overall, the available evidence suggests reduced tryptophan availability, altered KYN/TRP ratios, and an imbalance between neuroprotective and neurotoxic branches of the kynurenine pathway. In particular, reduced KYNA/QUIN and PIC (picolinic acid)/QUIN ratios have been associated with a shift toward a more neurotoxic metabolic profile, whereas longitudinal studies indicate that some of these abnormalities may change following antidepressant treatment. Representative clinical studies supporting these observations are summarized in Table 1.

## 7. The Tryptophan–Kynurenine Pathway in Alzheimer’s Disease

### 7.1. Neuroinflammation in Alzheimer’s Disease

AD is a progressive neurodegenerative disorder characterized by extracellular amyloid-β (Aβ) plaques, intracellular neurofibrillary tangles composed of hyperphosphorylated tau, synaptic dysfunction, neuronal loss, and progressive cognitive decline. Although amyloid and tau pathology remain central features of AD, increasing evidence indicates that chronic neuroinflammation is a major contributor to disease onset and progression [125,151,152]. Neuroinflammatory processes are not merely secondary responses to neuronal damage but active biological mechanisms capable of amplifying amyloid accumulation, tau phosphorylation, synaptic failure, and neurodegeneration [7].

Microglia, the resident immune cells of the CNS, play a central role in AD-related neuroinflammation. Under physiological conditions, microglia remove cellular debris, support synaptic remodeling, and participate in immune surveillance. In early AD, microglia may initially exert protective effects by clearing Aβ aggregates and limiting tissue injury. However, persistent exposure to Aβ, tau aggregates, oxidative stress, and peripheral inflammatory signals may drive microglia toward a chronically activated phenotype [23]. Chronically activated microglia release pro-inflammatory cytokines, including IL-1β, IL-6, TNF-α, and IFN-γ, together with ROS and nitric oxide. These mediators promote neuronal injury, impair synaptic function, and perpetuate local inflammatory responses [153].

Persistent activation of innate immune pathways contributes to amyloid deposition, tau pathology, synaptic dysfunction, and neuronal loss, supporting a central role for chronic neuroinflammation in AD progression [125,151].

Reactive astrocytes contribute to cytokine production, glutamatergic dysregulation, oxidative stress, and impaired neuronal repair in AD [154]. Together, activated microglia and astrocytes create a neuroinflammatory environment that favors progressive neuronal dysfunction.

Neuroinflammation promotes IDO activation and shifts tryptophan metabolism toward kynurenine production, linking immune dysregulation with oxidative stress, excitotoxicity, and cognitive decline [16].

### 7.2. Kynurenine Pathway Dysregulation and Cognitive Decline

Dysregulation of the kynurenine pathway may disturb the balance between neuroprotective metabolites such as KYNA and neurotoxic metabolites such as QUIN and 3-HK, thereby contributing to cognitive decline [17].

Inflammatory activation of IDO increases kynurenine production and favors neurotoxic metabolite generation by activated microglia [66,87].

In AD, chronic microglial activation tends to shift kynurenine metabolism toward the neurotoxic branch. Increased formation of 3-HK promotes oxidative stress, while QUIN enhances NMDA receptor activation and excitotoxicity [155]. These mechanisms impair synaptic plasticity, disrupt neuronal communication, and contribute to cognitive decline. Importantly, kynurenine pathway dysregulation in AD may exhibit regional heterogeneity rather than occurring uniformly throughout the brain. Alterations in kynurenine metabolism have been described in regions critically involved in memory, cognition, and neurodegeneration, including the hippocampus and frontal cortical areas, as well as subcortical structures such as the caudate nucleus and putamen [Maddison and Giorgini]. This regional heterogeneity may reflect differences in neuronal vulnerability, local microglial and astrocytic activation, glutamatergic signaling, and the regional distribution and activity of kynurenine pathway enzymes. The hippocampus may be particularly vulnerable to disturbances in the balance between neuroprotective and neurotoxic kynurenine metabolites because of its central role in learning and memory and its sensitivity to glutamatergic excitotoxicity [152,156]. Thus, local brain kynurenine metabolism may be more relevant to specific aspects of cognitive dysfunction than peripheral metabolite concentrations alone.

Clinical studies have reported altered kynurenine pathway metabolites in blood and cerebrospinal fluid of individuals with AD, although findings are not always consistent across studies. Some analyses suggest a shift toward kynurenine pathway activation in both peripheral and central compartments, while others indicate that peripheral kynurenine metabolite levels may not fully reflect CNS metabolism [157]. This discrepancy is important because AD-related kynurenine dysregulation may differ between plasma and cerebrospinal fluid, and local brain metabolism may be more relevant to cognitive decline than peripheral metabolite concentrations alone.

### 7.3. Quinolinic Acid-Mediated Neurotoxicity

Quinolinic acid is one of the most important neurotoxic metabolites of the kynurenine pathway and has received particular attention in neurodegenerative disorders. It is produced mainly by activated microglia and infiltrating macrophages and acts as an endogenous agonist of N-methyl-D-aspartate receptors [17]. Under physiological conditions, QUIN is an intermediate in NAD^+^ biosynthesis. However, when produced excessively during chronic inflammation, it becomes neurotoxic.

Oxidative stress generated by QUIN further amplifies neuronal vulnerability in AD. Lipid peroxidation, protein oxidation, and mitochondrial DNA damage contribute to progressive synaptic dysfunction and cognitive decline [87,158]. The consequences of QUIN-mediated neurotoxicity may also vary across brain regions because structures such as the hippocampus and neocortical areas are particularly vulnerable to disturbances in glutamatergic neurotransmission, oxidative stress, and excitotoxic injury [87,156].

Excessive QUIN production promotes NMDA receptor-mediated excitotoxicity, oxidative stress, glutamatergic dysregulation, and mitochondrial dysfunction, thereby contributing to synaptic failure and neuronal loss in AD [10,159].

QUIN also participates in neuroinflammatory amplification. It can activate microglia and stimulate additional inflammatory mediator release, thereby reinforcing the same inflammatory environment that promotes kynurenine pathway activation. This creates a pathological loop: inflammation activates IDO and kynurenine metabolism, kynurenine is converted into QUIN by activated microglia, and QUIN then promotes further excitotoxicity and inflammation. Neurons are highly dependent on mitochondrial energy production; therefore, QUIN-induced mitochondrial dysfunction may substantially contribute to neuronal loss observed in AD [87,159].

### 7.4. Interactions with Amyloid-β and Tau Pathology

The interaction between kynurenine pathway dysregulation and classical AD pathology is increasingly recognized as biologically relevant. Aβ accumulation can activate microglia and astrocytes, leading to increased release of inflammatory cytokines. These cytokines stimulate IDO activity and enhance kynurenine pathway flux, thereby increasing the production of downstream metabolites such as QUIN and 3-HK [160]. Thus, Aβ pathology may indirectly promote kynurenine pathway activation through neuroinflammation.

QUIN-induced oxidative stress and excitotoxicity may enhance amyloidogenic processing and tau phosphorylation, thereby linking kynurenine pathway activation to classical AD pathology [158,161].

Tau pathology may also be amplified by inflammatory signaling. Pro-inflammatory cytokines can activate intracellular pathways involved in tau phosphorylation, while microglial activation may facilitate tau propagation across neural networks [7]. Since kynurenine pathway activation is closely linked to inflammatory cytokine production, it may participate indirectly in tau-related neurodegeneration.

QUIN may further exacerbate Aβ-induced synaptic dysfunction through NMDA receptor overactivation and glutamatergic dysregulation [10].

The AhR may represent an additional link between tryptophan metabolism and AD pathology. Several kynurenine and microbial tryptophan metabolites can activate AhR, which regulates immune responses, oxidative stress, and cellular metabolism. Recent reviews suggest that dysregulated kynurenine–AhR signaling may contribute to AD-related inflammation and neurodegeneration, although this area remains under active investigation [162].

### 7.5. Evidence from Experimental and Clinical Studies

Experimental studies support the involvement of kynurenine pathway dysregulation in AD-related neurodegeneration. In cellular and animal models, inflammatory stimulation increases IDO and KMO activity, promotes QUIN production, and enhances oxidative stress and excitotoxicity [16]. Kynurenine pathway modulation has been shown to affect neuroinflammatory responses, synaptic function, and cognitive outcomes in experimental models, suggesting that the pathway may represent a therapeutic target [163].

Clinical evidence also supports an association between kynurenine metabolism and AD, although findings remain heterogeneous. A meta-analysis of central and peripheral kynurenine metabolites in AD suggested alterations in tryptophan metabolism, with evidence of kynurenine pathway involvement in both blood and cerebrospinal fluid. However, a more recent meta-analysis reported that AD dementia may be associated with lower blood levels of several kynurenine pathway metabolites, while cerebrospinal fluid KYNA may be increased [157]. These findings indicate that peripheral and central kynurenine metabolism may not follow identical patterns in AD.

Studies comparing plasma and cerebrospinal fluid kynurenines have also shown that peripheral metabolite concentrations do not always correlate strongly with central levels or with amyloid and tau biomarkers. This suggests that blood-based kynurenine markers should be interpreted cautiously and may need to be combined with cerebrospinal fluid biomarkers, neuroimaging, inflammatory markers, and cognitive assessments.

Overall, current evidence suggests that the tryptophan–kynurenine pathway contributes to AD through multiple interconnected mechanisms. Chronic neuroinflammation activates IDO and shifts tryptophan metabolism toward kynurenine production. Activated microglia then favor the generation of quinolinic acid and 3-HK, promoting excitotoxicity, oxidative stress, mitochondrial dysfunction, and synaptic damage. These processes interact with amyloid-β and tau pathology and may accelerate cognitive decline. Although further clinical studies are needed, the kynurenine pathway represents a promising mechanistic and therapeutic target in AD.

Clinical evidence supports alterations in kynurenine pathway metabolism in AD, although the reported changes vary according to disease stage and biological compartment. Representative studies evaluating kynurenine pathway metabolites and their clinical relevance in AD are summarized in Table 2.

## 8. Gut Microbiota Dysbiosis as a Shared Mechanistic Link Between Depression and Alzheimer’s Disease

Growing evidence indicates that gut microbiota dysbiosis may represent a common pathogenic factor contributing to both depression and AD. Alterations in microbial diversity and composition can disrupt the physiological balance of the microbiota–gut–brain axis, promoting intestinal barrier dysfunction, immune activation, chronic inflammation, and disturbances in tryptophan metabolism [10,14].

As discussed in Section 5, dysbiosis-associated impairment of intestinal barrier integrity may promote systemic immune activation and inflammatory signaling, thereby enhancing IDO activity and shifting tryptophan metabolism toward the kynurenine pathway [16,120]. Importantly, this mechanism may represent a shared biological link between gut microbial alterations and both depressive and neurodegenerative processes.

Kynurenine pathway dysregulation may therefore constitute a convergent molecular mechanism between depression and AD. In depression, altered tryptophan metabolism may contribute to reduced serotonergic availability and disturbances in neuroactive kynurenine metabolites, whereas in AD, increased formation of neurotoxic metabolites such as QUIN and 3-HK may contribute to oxidative stress, mitochondrial dysfunction, glutamatergic excitotoxicity, and progressive neuronal injury [17]. Despite differences in clinical expression, chronic inflammation and an altered balance between neuroprotective and neurotoxic kynurenine metabolites may therefore represent shared biological features of both disorders.

Beyond its effects on tryptophan metabolism, the gut microbiota may influence several biological processes relevant to both depression and AD, including immune homeostasis, SCFAs production, neurotransmitter signaling, and blood–brain barrier integrity [67]. Notably, studies in both conditions have reported reductions in potentially beneficial bacterial genera, including *Bifidobacterium*, *Lactobacillus*, *Faecalibacterium*, and *Roseburia*, together with enrichment of pro-inflammatory microbial taxa [159,171]. Although microbial signatures vary among studies and patient populations, these partially overlapping alterations support the possibility of shared microbiota-associated mechanisms contributing to systemic inflammation and kynurenine pathway dysregulation.

Taken together, current evidence suggests that gut microbiota dysbiosis may represent a common upstream modulator linking depression and AD. Rather than acting through a single pathway, microbial alterations may converge on several interconnected mechanisms, including impaired intestinal barrier function, chronic immune activation, altered microbial metabolite production, and dysregulation of tryptophan–kynurenine metabolism. These shared mechanisms may contribute to distinct clinical outcomes depending on disease-specific neuronal vulnerability, inflammatory status, aging-related changes, and underlying neuropathological processes.

Building on these shared mechanisms, we propose that kynurenine pathway dysregulation may represent a common molecular mechanism whose pathological consequences depend on the intensity, duration, anatomical localization, and biological context of pathway activation. In depression, systemic inflammation, stress-related HPA-axis activation, and altered gut microbial signaling may modify tryptophan availability and the balance between serotonergic metabolism and neuroactive kynurenine metabolites [88,138]. A shift toward reduced KYNA relative to QUIN may promote glutamatergic dysregulation, impaired synaptic plasticity, and altered function of neural circuits involved in mood and stress processing, thereby contributing predominantly to affective and cognitive symptoms [98,138,149]. In AD, by contrast, persistent activation of the kynurenine pathway in the context of aging, chronic neuroinflammation, microglial activation, and established neurodegenerative pathology may favor sustained production of 3-HK and QUIN, amplifying oxidative stress, mitochondrial dysfunction, excitotoxicity, and progressive synaptic and neuronal injury [89,156].

We therefore propose a context- and stage-dependent model in which kynurenine pathway dysregulation does not determine a specific disease phenotype by itself. Rather, its consequences may be shaped by the cellular and anatomical compartment involved, the relative balance between neuroprotective and neurotoxic metabolites, the duration of pathway activation, and the vulnerability of the affected neural networks [89,98,156]. Within this framework, depression may predominantly involve functional disturbances in neurotransmission and synaptic plasticity, whereas in AD persistent kynurenine pathway dysregulation may amplify neuroinflammatory and neurodegenerative processes [138,156]. These mechanisms are not mutually exclusive, and their overlap may provide a potential biological framework for understanding the coexistence of depressive and cognitive symptoms. This hypothesis should be tested in longitudinal studies integrating peripheral and central kynurenine metabolites, inflammatory markers, microbiome profiles, and disease-specific clinical and neuroimaging outcomes.

## 9. Peripheral-Central Decoupling of Kynurenine Pathway Metabolism and Implications for Biomarker Development

An important challenge in interpreting kynurenine pathway alterations in depression and AD is that metabolite concentrations measured in peripheral blood do not necessarily parallel those observed in the CNS. As summarized in Table 1 and Table 2, the direction and magnitude of kynurenine pathway abnormalities may differ according to the biological compartment examined. This is particularly evident for KYNA: in AD, a systematic review and meta-analysis reported increased KYNA concentrations in CSF but decreased concentrations in peripheral blood [164]. More broadly, clinical studies indicate that peripheral kynurenine metabolite concentrations may not fully reflect CNS kynurenine metabolism [157].

Several mechanisms may contribute to this peripheral–central decoupling. First, kynurenine pathway metabolites differ substantially in their ability to cross the blood–brain barrier. Kynurenine can enter the brain and thereby provide a peripheral substrate for central kynurenine metabolism, whereas downstream metabolites such as KYNA and QUIN have much more limited blood–brain barrier permeability [17,66]. Consequently, their CNS concentrations may depend substantially on local production rather than directly reflecting circulating concentrations. Second, kynurenine metabolism is characterized by marked cellular and tissue specialization. Within the CNS, astrocytes preferentially generate KYNA, whereas activated microglia are major producers of QUIN and other metabolites associated with the neurotoxic branch [87]. In contrast, peripheral kynurenine metabolism occurs in immune cells and multiple peripheral organs and is influenced by systemic inflammatory and metabolic signals [66,88]. Thus, the same systemic disturbance may produce different metabolic responses in peripheral and central compartments.

Inflammation and blood–brain barrier integrity may further modify this relationship. Peripheral inflammatory signals can alter blood–brain barrier permeability and activate endothelial, microglial, and astrocytic responses within the CNS [51]. At the same time, local neuroinflammation can regulate cerebral IDO activity and downstream metabolite production independently of circulating concentrations [87,89]. Disease stage may also be important: progressive neuroinflammation, changes in blood–brain barrier integrity, treatment exposure, aging, and metabolic comorbidities may dynamically alter the relationship between peripheral and central kynurenine metabolism. Peripheral and CSF measurements should therefore be regarded as complementary rather than interchangeable indicators of kynurenine pathway activity.

This compartmentalization has important implications for biomarker development. Easily accessible plasma or serum metabolites are attractive candidates for clinical biomarkers, but their interpretation as direct surrogates of cerebral kynurenine metabolism requires caution. Some metabolites may show meaningful peripheral–central associations; for example, studies in depression and AD have identified relationships between circulating and CSF kynurenines [142,169]. However, the direction of change may differ for other metabolites, particularly KYNA [168]. Accordingly, future biomarker studies should distinguish biomarkers of systemic kynurenine pathway activation from those intended to reflect CNS-specific processes. Whenever feasible, longitudinal studies integrating paired plasma and CSF measurements with inflammatory markers, neuroimaging, cognitive or psychiatric outcomes, and microbiome profiles may help determine which peripheral metabolites or metabolite ratios reliably predict central pathway activity and clinically relevant outcomes.

## 10. Therapeutic Perspectives

Growing evidence supporting the involvement of the microbiota–gut–brain axis and the tryptophan–kynurenine pathway in depression and AD has generated considerable interest in the development of novel therapeutic strategies targeting these interconnected biological systems. Unlike conventional approaches that primarily focus on symptom management, emerging interventions aim to modulate the underlying mechanisms linking inflammation, gut dysbiosis, altered tryptophan metabolism, and neurodegeneration [10,17].

One promising strategy involves the modulation of gut microbiota composition through dietary interventions, probiotics, prebiotics, synbiotics, and, more recently, fecal microbiota transplantation. Restoration of a healthy microbial ecosystem may improve intestinal barrier integrity, reduce systemic inflammation, increase SCFA production, and normalize tryptophan metabolism, thereby influencing both neurological and psychiatric outcomes [14,67]. Several clinical studies have reported beneficial effects of probiotic supplementation on depressive symptoms, cognitive performance, and inflammatory biomarkers, although larger randomized trials are still needed to establish their long-term efficacy [171].

Another important therapeutic target is the kynurenine pathway itself. Given the association between excessive pathway activation and the production of neurotoxic metabolites such as QUIN and 3-HK, pharmacological inhibition of key enzymes, including IDO and KMO, has attracted increasing attention [16]. Experimental studies suggest that limiting the formation of neurotoxic kynurenine metabolites while promoting the synthesis of neuroprotective compounds such as KYNA may reduce neuroinflammation, oxidative stress, and excitotoxic neuronal damage.

Because chronic inflammation represents a central mechanism linking gut dysbiosis, depression, and AD, anti-inflammatory therapies may also provide therapeutic benefit. Strategies aimed at reducing the production of pro-inflammatory cytokines or attenuating microglial activation have demonstrated promising results in experimental models and may indirectly normalize kynurenine pathway activity [126]. In addition, interventions targeting oxidative stress and mitochondrial dysfunction, including antioxidant compounds and lifestyle-based approaches such as physical exercise, have shown potential neuroprotective effects and may contribute to maintaining neuronal resilience [172].

The growing availability of microbiome profiling, metabolomics, and biomarker-based approaches also opens new opportunities for personalized medicine. Future therapeutic strategies may integrate individual microbial signatures, inflammatory profiles, and kynurenine metabolite patterns to identify patients most likely to benefit from specific interventions [66]. Although many of these approaches remain in the early stages of clinical development, current evidence suggests that targeting the microbiota–gut–brain axis and the tryptophan–kynurenine pathway may represent a promising avenue for the prevention and treatment of both depression and AD.

## 11. Sex-Dependent Modulation of the Kynurenine Pathway-Gut–Brain Axis

Sex is increasingly recognized as an important biological variable influencing the microbiota–gut–brain axis and kynurenine pathway regulation. Differences between females and males may emerge from the interaction of hormonal, immune, neuroendocrine, and metabolic factors, all of which can influence tryptophan availability and its distribution among serotonergic and kynurenine pathway branches. Sex-related differences in inflammatory signaling and hypothalamic–pituitary–adrenal (HPA) axis activity may also modify IDO- and TDO-dependent tryptophan metabolism, thereby affecting the balance between neuroprotective and neurotoxic kynurenine metabolites [64,66,89].

Clinical evidence in depression supports the relevance of sex-dependent kynurenine pathway alterations. In the longitudinal study by Nikkheslat et al. [148], adolescents at high risk for depression and those with MDD showed lower KYNA concentrations and reduced KYNA/QUIN ratios. Importantly, in females, a greater shift toward neurotoxic kynurenine metabolites was associated with persistent MDD during follow-up. These findings suggest that sex may influence not only kynurenine metabolite concentrations but also the prognostic significance of the balance between neuroprotective and neurotoxic branches of the pathway. This observation may partly explain the heterogeneity of kynurenine pathway findings across MDD populations and supports consideration of sex when interpreting kynurenine metabolites as potential biomarkers.

Sex-dependent modulation may extend upstream to the gut microbiota. Evidence from studies of depression indicates that gut microbial composition and its relationship with depressive symptoms can differ between females and males. A recent scoping review identified sex-related differences in microbial diversity and in the relative abundance of several bacterial taxa among individuals with depressive disorders, although the available clinical evidence remains limited and heterogeneous [173]. Such differences may be relevant to kynurenine metabolism because the gut microbiota regulates tryptophan availability, intestinal barrier integrity, microbial metabolite production, and systemic immune activation. Therefore, sex-specific microbial profiles could indirectly influence host tryptophan partitioning and kynurenine pathway activity, although direct studies simultaneously integrating sex, microbiome composition, and kynurenine metabolites remain scarce.

Sex-related differences are also relevant to AD. Biological sex may influence AD risk and progression through multiple mechanisms, including hormonal changes, immune and inflammatory responses, metabolic factors, genetic susceptibility, and age-related processes [174]. Importantly, emerging evidence indicates that kynurenine pathway alterations themselves may differ according to sex in AD. Knapskog et al. investigated cerebrospinal fluid concentrations of several kynurenine pathway metabolites in patients with symptomatic AD and cognitively unimpaired controls and identified sex-dependent differences in kynurenine metabolism. Most kynurenine metabolites were higher in men than in women within the AD group, whereas comparable sex differences were not observed among cognitively unimpaired individuals. Moreover, sex-specific associations were identified between KYNA, the KYNA/QUIN ratio, and neopterin, supporting an interaction between biological sex, kynurenine metabolism, and neuroinflammatory activity in AD [175].

These observations suggest that sex should be considered a biological modifier rather than a simple demographic covariate within the microbiota–gut–brain–kynurenine framework. In depression, current evidence indicates that sex may influence the balance between neuroprotective and neurotoxic kynurenine metabolites and their association with disease persistence [148], whereas in AD, sex-dependent relationships between kynurenine metabolites and neuroinflammatory markers are beginning to emerge [175]. At the same time, sex-related variation in gut microbial composition may provide an additional upstream source of heterogeneity [173]. However, evidence directly connecting all three components—sex, gut microbiota, and kynurenine metabolism—within the same clinical populations remains limited. Future studies should therefore stratify participants according to sex and, where appropriate, hormonal status and age, while simultaneously integrating microbiome, inflammatory, and kynurenine pathway measurements. Such approaches may improve interpretation of KP biomarkers and support more individualized therapeutic strategies in both MDD and AD.

## 12. Future Directions and Research Gaps

Despite substantial advances in understanding the microbiota–gut–brain axis and the tryptophan–kynurenine pathway, several important questions remain unresolved. Although growing evidence supports the involvement of gut microbiota dysbiosis and kynurenine pathway dysregulation in both depression and AD, the causal relationships between these processes have not yet been fully established. Most available studies are cross-sectional and therefore cannot determine whether microbial and metabolic alterations represent initiating factors, compensatory responses, or consequences of disease progression [10,14].

Another major challenge concerns the considerable heterogeneity observed among studies investigating the gut microbiome. Differences in population characteristics, dietary habits, geographical location, sequencing methodologies, and analytical approaches often result in inconsistent findings regarding specific microbial signatures associated with depression and AD [171]. Standardized protocols and large multicenter studies will be necessary to identify reproducible microbiota-based biomarkers with clinical utility.

Similarly, although several kynurenine pathway metabolites have been proposed as potential biomarkers of neuroinflammation and disease severity, their clinical applicability remains limited. Peripheral measurements do not always accurately reflect central nervous system metabolism, and considerable variability exists among studies regarding metabolite concentrations and metabolite ratios [17]. Future investigations should integrate metabolomics, neuroimaging, cerebrospinal fluid biomarkers, and microbiome profiling to obtain a more comprehensive understanding of disease-associated biological alterations.

An additional area requiring further investigation is the development of targeted therapeutic strategies. While preclinical studies have demonstrated promising effects of microbiota modulation, IDO inhibition, KMO inhibition, and anti-inflammatory interventions, robust clinical evidence remains scarce. Large randomized controlled trials are needed to evaluate the safety, efficacy, and long-term benefits of these approaches in patients with depression and AD [16].

Finally, advances in systems biology and precision medicine may facilitate the identification of biologically distinct patient subgroups characterized by specific inflammatory, microbial, or metabolic profiles. Integrating microbiome analysis with genetic, immunological, and metabolomic data may ultimately enable personalized interventions targeting the microbiota–gut–brain axis and the kynurenine pathway [66]. Such approaches have the potential to improve early diagnosis, optimize treatment selection, and reduce the burden of both psychiatric and neurodegenerative disorders.

## 13. Conclusions

Depression and AD are complex disorders that represent major challenges for public health worldwide. Although traditionally considered distinct clinical entities, growing evidence indicates that they share several biological mechanisms, including chronic inflammation, oxidative stress, mitochondrial dysfunction, altered neurotransmission, and gut microbiota dysbiosis. These interconnected processes contribute to neuronal dysfunction and may accelerate both mood disturbances and cognitive decline.

The microbiota–gut–brain axis has emerged as an important framework for understanding how peripheral physiological changes can influence brain function. Within this axis, the tryptophan–kynurenine pathway represents a key molecular link connecting gut microbiota, immune activation, and central nervous system processes. Dysregulation of this pathway may reduce serotonin availability while increasing the production of neurotoxic metabolites such as QUIN and 3-HK, thereby promoting neuroinflammation, oxidative stress, and neuronal damage.

Based on the evidence integrated in this review, we propose that the pathogenic relevance of the kynurenine pathway is determined not simply by its overall activation, but by the context-dependent redistribution of tryptophan metabolism among serotonergic, neuroprotective, and neurotoxic branches. This integrative microbiota–immune–tryptophan model positions the kynurenine pathway as a dynamic molecular interface through which shared upstream disturbances, including intestinal dysbiosis and immune activation, may contribute to distinct but partially overlapping manifestations in depression and AD. Accordingly, the relative distribution of kynurenine metabolites, rather than changes in individual metabolites alone, may provide a more informative framework for understanding disease heterogeneity and for identifying potential biomarkers and therapeutic targets.

Building on this integrative model, several experimentally testable hypotheses can be proposed. First, we hypothesize that the relative balance between neuroprotective and neurotoxic kynurenine metabolites, rather than individual metabolite concentrations, may better identify biologically distinct subgroups of patients with depression or AD. Second, specific dysbiosis-associated inflammatory profiles may promote IDO-dependent redistribution of tryptophan metabolism toward the kynurenine pathway and a shift toward neurotoxic metabolites. Third, the clinical consequences of this metabolic shift may depend on biological context, including sex, age, inflammatory status, disease stage, and peripheral versus central kynurenine pathway activity. These hypotheses could be tested in longitudinal studies integrating gut microbiome profiling, inflammatory biomarkers, plasma and cerebrospinal fluid kynurenine metabolites, and disease-specific clinical or neuroimaging outcomes.

These hypotheses also provide a framework for future diagnostic and therapeutic studies. We propose that multimodal biomarker panels combining microbial signatures, inflammatory mediators, and kynurenine metabolite ratios may provide greater diagnostic and prognostic value than individual biomarkers alone. From a therapeutic perspective, interventions targeting gut dysbiosis, inflammation, or specific kynurenine pathway enzymes may be most effective in biologically defined patient subgroups characterized by corresponding microbiota–immune–kynurenine profiles. Prospective studies should therefore determine whether changes in these integrated profiles precede clinical progression or predict therapeutic response, while randomized intervention studies should establish whether their normalization is accompanied by clinical improvement. Testing these predictions may help translate the microbiota–immune–tryptophan model from a mechanistic framework into biomarker-guided and personalized strategies for the diagnosis, prognosis, and treatment of depression and AD.

## Figures and Tables

**Table 1:** Clinical evidence linking kynurenine pathway dysregulation to major depressive disorder.

No.	Study Design	Biological Sample	Kynurenine Pathway Metabolites/Ratios	Key Findings	Clinical Relevance	References
1	Case–control; current MDD, remitted MDD, healthy controls	Plasma	TRP, KYN, KYNA, QUIN; KYNA/QUIN	KYNA/QUIN ratio was reduced in both current and remitted MDD	Suggests a persistent imbalance toward relatively greater neurotoxic KP activity, even during remission	Savitz et al., 2015 [141]
2	Human experimental inflammatory challenge	Plasma	TRP, KYN, KYNA, QUIN and related metabolites	Acute inflammation altered kynurenine metabolism, and changes were associated with inflammation-induced depressed mood	Provides experimental human evidence linking immune activation, KP metabolism and depressive symptoms	Kruse et al., 2019 [142]
3	Clinical study; 72 unmedicated depressed patients	Plasma and CSF	TRP, KYN, KYNA, QUIN, KYN/TRP	Plasma TNF correlated with KYN and KYN/TRP; peripheral KP activation was related to central KP metabolites and greater depression severity/anhedonia	Strong evidence connecting peripheral inflammation with central kynurenine metabolism in depression	Haroon et al., 2020 [143]
4	Antidepressant treatment study; escitalopram vs. desvenlafaxine	Plasma	Serotonin and multiple KP metabolites	Baseline and treatment-related changes in serotonin/KP metabolites differed according to antidepressant response	Suggests potential value of KP metabolites as predictors or correlates of treatment response	Sun et al., 2020 [144]
5	MDD patients vs. healthy controls; peripheral and central assessments	Plasma and CSF	PIC, KYNA, QUIN, KYNA/QUIN, PIC/QUIN and others	MDD was associated with lower PIC, KYNA/QUIN and PIC/QUIN ratios, with increased QUIN; abnormalities were also related to metabolic disturbances	Supports a shift from neuroprotective toward neurotoxic KP metabolism in MDD	Paul et al., 2022 [145]
6	Drug-naïve first-episode MDD; adult and adolescent cohorts	Plasma	TRP-related and monoamine metabolites	Disturbances in neurotransmitter metabolism differed between adult and adolescent MDD	Indicates age-dependent heterogeneity in TRP-related metabolic abnormalities	Wang et al., 2022 [146]
7	Longitudinal treatment study; depressed patients undergoing 6-week multimodal rehabilitation	Blood	KYN and downstream KP metabolites	KP metabolite changes were associated with therapeutic response over treatment	Supports dynamic KP changes as potential treatment-response biomarkers rather than static diagnostic markers	Reininghaus et al., 2024 [147]
8	MDD with type 2 diabetes vs. MDD alone	Blood	TRP, QUIN/TRP, serotonin; TNF-α and IL-6	TNF-α significantly modified associations involving QUIN/TRP and serotonin; QUIN/TRP was higher in comorbid MDD/T2DM	Shows that inflammatory/metabolic comorbidity can markedly influence KP abnormalities in depression	Okamoto et al., 2024 [148]
9	Longitudinal adolescent cohort; low risk, high risk and MDD groups; 3-year follow-up	Plasma	KYN, KYNA, QUIN, 3-HK and ratios	High-risk and MDD adolescents had lower KYNA and KYNA/QUIN; in females, greater diversion toward neurotoxic metabolites predicted persistent MDD	Important evidence for sex-specific and prognostic KP alterations in adolescent depression	Nikkheslat et al., 2025 [149]
10	Longitudinal antidepressant-treatment study	Plasma	KYN pathway metabolites	Several KP metabolites increased after 6 months of antidepressant treatment, with many approaching control levels	Suggests that KP dysregulation is at least partly state-dependent and modifiable by treatment	Colle et al., 2025 [150]

MDD—Major Depressive Disorder; TRP—tryptophan; KYN—kynurenine; KP—kynurenine pathway; KYNA—kynurenic acid; QUIN—quinolinic acid; KYNA/QUIN—Kynurenic acid/quinolinic acid ratio; 3-HK—3-hydroxykynurenine; PIC—picolinic acid; KYN/TRP ratio—kynurenine/tryptophan ratio; TNF-α—tumor necrosis factor α; IL-6—interleukine 6; KP—Kynurenine pathway; PIC/QUIN—Picolinic acid/quinolinic acid ratio; T2DM—type 2 diabetes mellitus.

**Table 2:** Clinical Evidence of Tryptophan–Kynurenine Pathway Alterations in Alzheimer’s Disease.

No.	Study Design	Biological Sample	Kynurenine Pathway Metabolites/Ratios	Key Findings	Clinical Relevance	References
1	Case–control; 34 patients with AD and 18 age-matched controls	Plasma	TRP, KYN, KYNA, 3-HK, AA, QUIN	AD patients showed lower TRP and KYNA and markedly higher QUIN; cognitive performance correlated positively with KYNA and inversely with QUIN	Provides early clinical evidence of peripheral KP dysregulation and an altered balance between neuroprotective and neurotoxic metabolites in AD	Gulaj et al., 2010 [164]
2	Case–control; 65 histopathologically confirmed AD patients and 65 cognitively screened controls	Plasma	TRP and multiple kynurenines, including KYN, KYNA, XA and QUIN	Several circulating kynurenines were lower in AD; elevated QUIN was associated with poorer cognition in older AD patients	Supports an association between KP alterations and cognitive impairment, while also showing heterogeneity of peripheral KP changes in AD	Giil et al., 2017 [165]
3	Longitudinal study; 155 patients with mild dementia, including 90 AD and 65 Lewy body dementia; 5-year follow-up	Serum	TRP, KYN, KYNA and other kynurenines; KYN/TRP and KYNA/KYN ratios	KYN showed a complex relationship with cognition; higher KYNA/KYN-related activity was associated with neuropsychiatric symptoms, particularly hallucinations	Suggests that KP metabolites may be related not only to cognition but also to neuropsychiatric manifestations of dementia	Solvang et al., 2019 [166]
4	Case–control study; AD and other neurodegenerative disorders compared with cognitively healthy controls	CSF	KYNA	CSF KYNA concentrations were significantly higher in AD than in healthy controls and other neurodegenerative disease groups	Suggests disease-associated central KP alterations and potential diagnostic relevance of CSF KYNA	González-Sánchez et al., 2020 [167]
5	Systematic review and meta-analysis; 22 studies, 1356 participants (664 AD, 692 cognitively normal)	Peripheral blood and CSF	TRP, KYN, KYN/TRP, KYNA, 3-HK, QUIN	Peripheral TRP was reduced and KYN/TRP increased; CSF KYNA was increased whereas peripheral KYNA was decreased; no significant overall difference in QUIN	Demonstrates compartment-specific KP alterations and cautions against extrapolating peripheral metabolite concentrations directly to the CNS	Fernandes et al., 2023 [168]
6	Memory-clinic cohort across the cognitive spectrum; 138 patients	Plasma and CSF	TRP, KYN, KYNA, 3-HK, XA, AA, 3-HAA, QUIN, PIC	Plasma concentrations of several kynurenines correlated with corresponding CSF levels; associations were also identified between selected CSF kynurenines and AD-related biomarkers	Supports further investigation of peripheral kynurenines as less-invasive indicators of central KP metabolism and AD pathology	Bakker et al., 2023 [169]
7	Longitudinal case–control; 311 AD patients and 105 cognitively unimpaired controls	CSF	KYNA, PIC, QUIN and other KP metabolites	AD patients had higher KYNA and PIC; higher KYNA was associated with tau pathology but also with slower dementia progression; neurotoxic kynurenines were not increased	Suggests that activation of the neuroprotective KP branch may represent an adaptive response and that KYNA could have prognostic relevance	Knapskog et al., 2023 [170]
8	Systematic review and meta-analysis of cognitive impairment and dementia	Blood and CSF	Multiple KP metabolites and ratios	Demonstrated KP dysregulation across cognitive impairment and dementia, while emphasizing heterogeneity according to disease stage and biological compartment	Strengthens evidence for KP involvement in cognitive impairment while highlighting the need for compartment- and disease-stage- specific interpretation	Choe et al., 2026 [157]

AD—Alzheimer’s disease; AA—anthranilic acid; XA—xanthurenic acid; CSF—cerebrospinal fluid; TRP—tryptophan; KYN—kynurenine; KP—kynurenine pathway; KYNA—kynurenic acid; QUIN—quinolinic acid; KYNA/QUIN—Kynurenic acid/quinolinic acid ratio; 3-HK—3-hydroxykynurenine; PIC—picolinic acid; KYN/TRP ratio—kynurenine/tryptophan ratio; TNF-α—tumor necrosis factor α; IL-6—interleukine 6; KP—Kynurenine pathway; PIC/QUIN—Picolinic acid/quinolinic acid ratio; 3-HAA—3-hydroxyanthranilic acid.

## Data Availability

No new data were created or analyzed in this study. Data sharing is not applicable to this article.

## Abbreviations

3-HAA	3-hydroxyanthranilic acid
3-HAO	3-hydroxyanthranilate 3,4-dioxgenase
5-HIAA	5-hydroxyindoleacetic acid
5-HTP	5-hydroxy-L-tryptophan
AADC	aromatic L-amino acid decarboxylase
AANAT	arylalkylamine N-acetyltransferase
ACMS	2-amino-3-carboxymconate semialdehyde
ACTH	adrenocorticotropic hormone
AD	Alzheimer’s disease
AgRP	agouti-related peptide
AhR	aryl hydrocarbon receptor
ANS	autonomic nervous system
ASMT	acetylserotonin O-methyltransferase
Aβ	amyloid-β
BBB	blood–brain barrier
CCK	cholecystokinin
CNS	central nervous system
CRH	corticotropin-releasing hormone
CSF	cerebrospinal fluid
ENS	enteric nervous system
FXR	Farnesoid X Receptor
GABA	gamma-aminobutyric acid
GALT	gut-associated lymphoid tissue
GBA	gut–brain axis
GLP-1	glucagon-like peptide-1
GPR41	G-protein-coupled receptors 41
GPR43	G-protein-coupled receptors 43
HDAC	histone deacetylase
3-HK	3-hydroxykynurenine
HPA axis	hypothalamic–pituitary–adrenal axis
5-HT	5-hydroxytryptamine
IAld	indole-3-aldehyde
IDO	indoleamine 2,3-dioxygenase
IFN-γ	interferon-gamma
IL-10	interleukin-10
IL-1β	interleukin-1β
IL-6	interleukin-6
IPA	indole-3-propionic acid
JAK/STAT1	JANUS kinase/Signal Transducer and Activator of Transcription 1
KATs	indoleamine 2,3-dioxygenase
KMO	kynurenine 3-monooxygenase
KP	kynurenine pathway
KYN/TRP	kynurenine-to-tryptophan ratio
KYNA	kynurenic acid
LAT1	large amino acid transporter 1
LNAAs	large neutral amino acids
LPS	lipopolysaccharides
MAMPs	microbial-associated molecular patterns
MAO-A	monoamine oxidase A
NAD+	nicotinamide adenine dinucleotide
NF-κB	nuclear factor kappa B
NTS	nucleus tractus solitarius
POMC	proopiomelanocortin
PYY	peptide YY
QUIN	quinolinic acid
SCFAs	short-chain fatty acids
TDO	tryptophan 2,3-dioxygenase
TGR5	Takeda G protein-coupled receptor 5
TLR4	toll-like receptor 4
TLRs	toll-like receptors
TNF-α	tumor necrosis factor-alpha
TPH	tryptophan hydroxylase
TPH1	tryptophan hydroxylase 1
TPH2	tryptophan hydroxylase 2
Tregs	regulatory T cells
XA	xanthurenic acid
ZO-1	zonula occludens-1
